# A tyrosine phosphoregulatory system controls exopolysaccharide biosynthesis and biofilm formation in *Vibrio cholerae*

**DOI:** 10.1371/journal.ppat.1008745

**Published:** 2020-08-25

**Authors:** Carmen Schwechheimer, Kassidy Hebert, Sarvind Tripathi, Praveen K. Singh, Kyle A. Floyd, Elise R. Brown, Monique E. Porcella, Jacqueline Osorio, Joseph T. M. Kiblen, Fernando A. Pagliai, Knut Drescher, Seth M. Rubin, Fitnat H. Yildiz

**Affiliations:** 1 Department of Microbiology and Environmental Toxicology, University of California—Santa Cruz, Santa Cruz, California, United States of America; 2 Department of Chemistry and Biochemistry, University of California—Santa Cruz, Santa Cruz, California, United States of America; 3 Max Planck Institute for Terrestrial Microbiology, Marburg, Germany; 4 Department of Physics, Philipps-Universität Marburg, Marburg, Germany; University of Washington, UNITED STATES

## Abstract

Production of an extracellular matrix is essential for biofilm formation, as this matrix both secures and protects the cells it encases. Mechanisms underlying production and assembly of matrices are poorly understood. *Vibrio cholerae*, relies heavily on biofilm formation for survival, infectivity, and transmission. Biofilm formation requires *Vibrio* polysaccharide (VPS), which is produced by *vps* gene-products, yet the function of these products remains unknown. Here, we demonstrate that the *vps* gene-products *vpsO* and *vpsU* encode respectively for a tyrosine kinase and a cognate tyrosine phosphatase. Collectively, VpsO and VpsU act as a tyrosine phosphoregulatory system to modulate VPS production. We present structures of VpsU and the kinase domain of VpsO, and we report observed autocatalytic tyrosine phosphorylation of the VpsO C-terminal tail. The position and amount of tyrosine phosphorylation in the VpsO C-terminal tail represses VPS production and biofilm formation through a mechanism involving the modulation of VpsO oligomerization. We found that tyrosine phosphorylation enhances stability of VpsO. Regulation of VpsO phosphorylation by the phosphatase VpsU is vital for maintaining native VPS levels. This study provides new insights into the mechanism and regulation of VPS production and establishes general principles of biofilm matrix production and its inhibition.

## Author summary

The biofilm life style protects microbes from a plethora of harm, to increase their survival and pathogenicity. Exopolysaccharides are the essential glue of the microbial biofilm matrix, and loss of this glue negates biofilm formation and renders cells more sensitive to antimicrobial agents. Here, we show that a tyrosine phosphoregulatory system controls the biosynthesis and abundance of *Vibrio* exopolysaccharide (VPS), an essential biofilm component of the pathogen *Vibrio cholerae*. The phosphorylation state of the tyrosine autokinase VpsO, mediated by the tyrosine phosphatase VpsU, directly modulates VPS production and also affects the kinase’s own degradation, to regulate VPS production. This study provides new insights into the mechanisms of *V*. *cholerae* biofilm formation and consequently ways to combat pathogens more broadly, due to conservation of tyrosine phosphoregulatory systems among exopolysaccharide producing bacteria.

## Introduction

Biofilm formation plays a fundamental role in the infection cycle of many bacterial pathogens. Biofilm formation by *Vibrio cholerae*, the causal organism of the diarrheal disease cholera, increases the ability of the organism to survive in the aquatic habitats and enhances its transmission and infectivity [1]. A major component of the *V*. *cholerae* biofilm matrix is a self-produced exopolysaccharide known as *Vibrio* polysaccharide (VPS) [2,3]. *V*. *cholerae* produces two types of VPS. The major variant has a repeating unit of [→4)-α-L-Gul*p*NAcAGly3OAc-(1→4)-β-D-Glc*p*-(1→4)-α-D-Glc*p*-(1→4)-α-D-Gal*p-*(1→]_n_, whereas the minor variant (~20%) has α-D-GlcNAc instead of α-D-Glc [3–5]. VPS production is essential for the development of biofilm architecture, which is stabilized by interactions between VPS and the protein components of the biofilm matrix [6–9]. VPS contributes to the *in vivo* fitness of *V*. *cholerae* [4], provides protection from oxidative stress [3,10,11], and protects *V*. *cholerae* cells from Type 6 secretion system attacks [12] and protozoan grazing [13–17]. Finally, VPS contributes to the formation of biofilm aggregates of conditionally viable environmental cells (CVECs) that are critical for *V*. *cholerae* transmission [18]. Cells unable to produce VPS cannot form CVECs and also exhibit reduced intestinal colonization [19].

**Fig 1:**
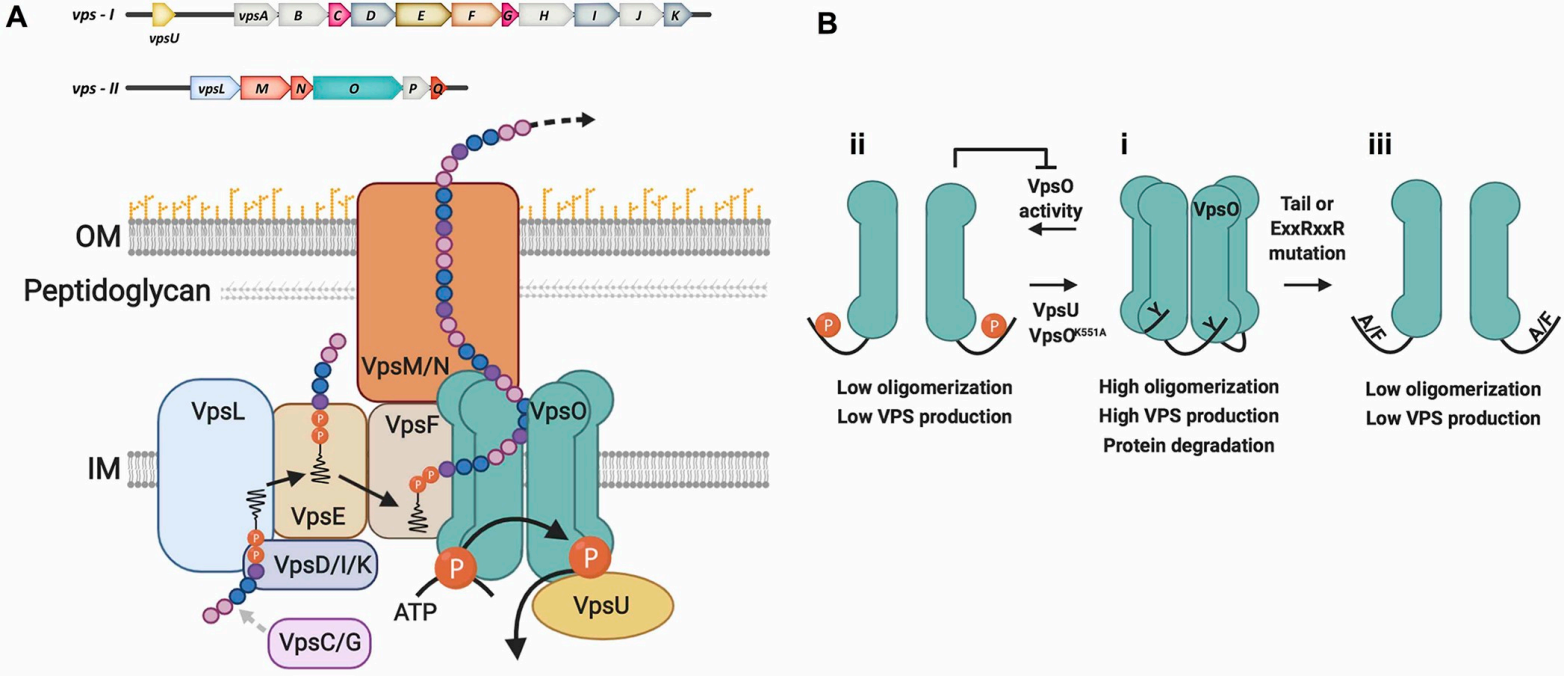
A tyrosine phosphoregulatory system controls production of VPS to modulate *V*. *cholerae* biofilm formation. **(A)** Biosynthesis of VPS polysaccharide depends on the *vps* gene products. The 18 *vps* genes are clustered in two regions on the large chromosome of *V*. *cholerae* O1 El Tor. The *vps-*I cluster contains *vpsU* (VC0916) and *vpsA-K* (VC0917-27, 11464 nucleotides); *vpsU* is separated from *vpsA* by 501 nucleotides. The *vps-*II cluster [*vpsL-Q*, (VC0934-9)] is 6550 nucleotides [4]. Based on homology with other exopolysaccharide and capsule biosynthesis systems, we propose that VPS is synthesized by formation and polymerization of individual repeat units, followed by transfer of the resulting polymer across the outer membrane. **(B)** A model for the VpsO and VpsU tyrosine phosphoregulatory system. VpsO is a tyrosine kinase, which utilizes a catalytic lysine residue (K551) to autophosphorylate multiple tyrosine residues in the C-terminal tail of its cytoplasmic kinase domain. The low-molecular-weight tyrosine phosphatase, VpsU, dephosphorylates the C-terminal tail of VpsO. Cycling between high and low levels of the C-terminal tyrosine-cluster phosphorylation regulates VPS production. (i) The unphosphorylated high oligomerization state of VpsO leads to high VPS production and increased proteolytic degradation of VpsO. (ii) In the native phosphoregulatory system, autokinase activity leads to VpsO phosphorylation, higher order oligomer dissociation, and a reduction in VPS production. VpsU dephosphorylates VpsO to shift the equilibrium towards the low phosphorylation and high oligomerization state and increased VPS production. The catalytically inactive version, VpsO^K551A^, results in the complete loss of phosphorylation, high oligomerization, and hyper VPS production. VpsO C-terminal tail phosphorylation also inhibits catalytic activity and may attenuate the transition to the dissociated state. (iii) Impairing oligomerization of VpsO, either by mutation of the cytoplasmic ExxRxxR motif or by mutation of the C-terminal tail tyrosines to alanines or phenylalanine (A/F), leads to low VPS production. Most likely this is the case because the tyrosine hydroxyl groups are important for affinity of the C-terminal tail for the neighboring BY-kinase subunit, and mutation to alanine or phenylalanine breaks this interaction similar to tyrosine phosphorylation. Thus, the tyrosine mutations mimic the VpsO tyrosine phosphorylated state in their effects on structure and function.

Production of VPS requires the 18 *vps* genes that are clustered in two regions on the large chromosome of *V*. *cholerae* O1 El Tor. The genes *vpsU* (VC0916) and *vpsA-K* (VC0917-27) are located in the *vps-I* cluster, and *vpsL-Q* (VC0934-9) are located in the *vps-II* cluster (Fig 1A) [3,4]. However, the precise role of each *vps* gene product is not known. Bioinformatic analysis suggests that VPS biosynthesis occurs in a Wzy-polymerase and Wzx-flippase dependent assembly pathway (Fig 1A) [4]. *vpsO* (*VC0937*) and *vpsU* (*VC0916*) have sequence homology to the tyrosine kinase and low-molecular-weight protein tyrosine phosphatase, respectively, in the Wzy/Wzx-dependent capsule/exopolysaccharide assembly pathways in other bacterial species [20,21]. Our earlier work showed that loss of *vpsO* leads to the complete absence of VPS production, whereas the loss of *vpsU* reduces VPS production [4]; however, the molecular details of how this system controls VPS biosynthesis and in turn biofilm formation are not known.

Bacterial tyrosine kinases (BY-kinase) and tyrosine phosphatases are widely distributed in Gram-positive and Gram-negative bacteria [22]. Tyrosine phosphorylation plays an integral role in production of bacterial surface glycans involved in extracellular polysaccharide matrix and capsule production [21,23–32]. Typically, Gram-negative BY-kinases are transmembrane proteins that possess a periplasmic regulatory domain and a cytoplasmic kinase domain with a C-terminal tail enriched in tyrosine residues [23]. The C-terminal tyrosine-rich tail is the predominant target of autophosphorylation [28,33–36]. Tyrosine phosphorylation has either a positive or negative impact on polysaccharide production depending on the species and system, and it has been implicated in biofilm formation [24,29,32,35,37–41]. However, the molecular underpinnings of the role of BY-kinases in extracellular polysaccharide biosynthesis and the consequences of protein tyrosine phosphorylation in biofilm formation remain unclear.

In this study, we have identified and characterized the role of a tyrosine phosphoregulatory system in *V*. *cholerae* VPS production and biofilm formation (Fig 1A). We found that VpsO and VpsU have respective kinase and phosphatase functions, and we have determined crystal structures of VpsU and the VpsO catalytic domain. Structural data indicate distinct differences between VpsO and other known BY-kinases. Tyrosine phosphorylation of the VpsO C-terminal tail results in a conformational change that suggests inhibition of further catalytic activity. Further, we found that tyrosine phosphorylation enhances stability of VpsO. We identified the tyrosine phosphorylation sites of VpsO, and determined the specific residues that are critical for regulation of VPS production and biofilm formation. Autophosphorylation of the VpsO C-terminal tail disrupts its oligomerization and inhibits VPS production (Fig 1B). We found that the VpsU tyrosine phosphatase controls VPS production through regulation of the VpsO phosphorylation state. Together, our data suggest that the phosphorylation state of the C-terminal tail of VpsO, modulated by autokinase and VpsU phosphatase activity, regulates the functional state of VpsO to control VPS production and biofilm formation in *V*. *cholerae*. Lastly, BY-kinases are considered novel targets for antibiotics due to structural difference from their eukaryotic counterparts, and here we demonstrate that the loss of VpsO oligomerization renders *V*. *cholerae* cells sensitive to antibiotics.

## Results

### VpsO is a tyrosine kinase with autocatalytic activity

To gain insight into the mechanisms regulating VPS production, we first analyzed tyrosine kinase activity of VpsO, which is predicted from VpsO homology to known BY-kinases. Sequence analysis showed that the 737-amino-acid VpsO protein is composed of an N-terminal periplasmic domain (amino acids 45–448), two transmembrane helices, a C-terminal cytoplasmic BY-kinase domain (amino acids 473–737), and a C-terminal tail containing six tyrosine residues (Y717, Y720, Y721, Y723, Y726, and Y727). Based on homology to other BY-kinases, we hypothesized that VpsO residues 503–737 form the minimal kinase domain, while residues 473–503 comprise an N-terminal extension that is required for activity in some other BY-kinases [42]. We cloned and purified two C-terminal cytoplasmic domains starting at residue 473 and at residue 503 (VpsO-473 and VpsO-503), and we evaluated VpsO tyrosine kinase activity by determining autophosphorylation in the presence of [γ-^32^P]-ATP. We found that both constructs were phosphorylated at similar levels (S1A Fig), and we used the minimal VpsO-503 kinase domain in subsequent experiments.

**Fig 2:**
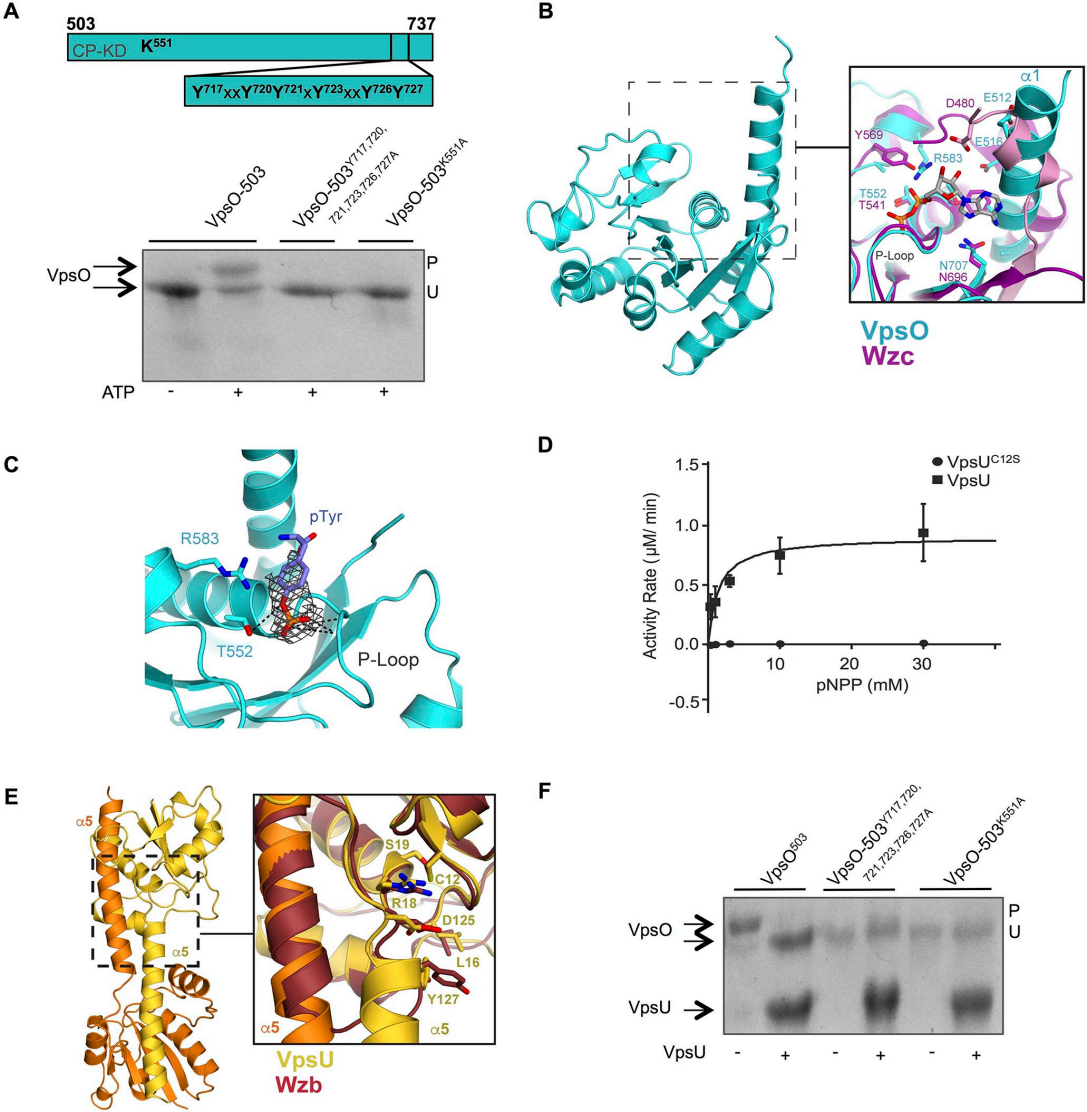
Biochemical and structural characterization of the kinase domain of VpsO and its cognate phosphatase VpsU. **(A**) Diagram of VpsO-503 and the position of the catalytic lysine residue (K551) and the potential C-terminal tail phosphorylation sites. VpsO kinase activity was evaluated by the Phos-Tag SDS-PAGE migration pattern of VpsO-503 and point mutants; n≥2. VpsO was incubated with 10 mM MgCl_2_ in the presence and absence of ATP for 30 minutes at 37°C, prior to the Phos-Tag electrophoresis. P refers to the tyrosine phosphorylated state and U refers to the unphosphorylated state. Coomassie gel staining was used for protein visualization. In this experiment, the purified proteins were prepared such that they were initially unphosphorylated. CytoPlasmic Kinase Domain (CP-KD) **(B)** The 2.9-Å crystal structure of the VpsO^E519A, R522A, R525A^ kinase domain (cyan) compared to the structure of the Wzc kinase domain (magenta). Residues at the N-terminus of α1 in VpsO likely replace the N-terminal extension in Wzc (pink) in binding adenosine. The ADP is modeled in from the Wzc structure (PDB: 3LA6). **(C)** Electron density in the active site of VpsO modeled with a phosphotyrosine. The electron density is shown only near the phosphotyrosine from a 2mf_o_−Df_c_ simulated-annealing composite omit map contoured at 1σ. **(D)** Determination of the catalytic rates of VpsU and VpsU^C12S^ in the presence of the generic substrate pNPP; n≥4. **(E)** VpsU crystal structure aligned with the *E*. *coli* homolog Wzb (PDB: 2WJA). **(F)** Phos-Tag SDS-PAGE migration pattern of WT and mutated versions of VpsO-503 proteins with or without prior incubation with VpsU for 30 minutes at 37°C; n≥2. P refers to the phosphorylated state and U refers to the unphosphorylated state. Coomassie gel staining was used for protein visualization. The WT VpsO-503 in this experiment is initially heterogeneously phosphorylated following recombinant expression and purification.

To determine whether the tyrosine-rich C-terminal tail of VpsO contains sites of tyrosine phosphorylation, we purified a VpsO kinase domain in which all six C-terminal tail tyrosine residues were mutated to alanine residues (VpsO-503^Y717A, Y720A, Y721A, Y723A, Y726A, Y727A^). We also purified a domain in which the predicted active site lysine residue (K551) was replaced with an alanine (VpsO-503^K551A^). We performed an *in vitro* phosphorylation assay with each domain and analyzed the reaction products using a Phos-Tag gel, in which phosphorylation of a protein induces its slower migration. We found that upon incubation with ATP the wild-type version of the VpsO-503 domain underwent a mobility shift consistent with autophosphorylation, while both the VpsO-503^Y717A, Y720A, Y721A, Y723A, Y726A, Y727A^ and the VpsO-503^K551A^ mutated domains showed no mobility shift and therefore no autophosphorylation in this assay (Fig 2A). We further confirmed tyrosine phosphorylation in VpsO-503 and the absence of tyrosine phosphorylation in VpsO-503^K551A^ using electrospray ionization mass spectrometry (S1B Fig). Collectively, these data show that the VpsO C-terminal domain has tyrosine autokinase activity dependent upon lysine 551 and that VpsO autophosphorylates multiple tyrosine residues in the C-terminal tail.

We next set out to determine the structure of the cytoplasmic kinase domain of VpsO. We found that the only version of VpsO-503 that we could crystallize was VpsO-503^E519A, R522A, R525A^, which contains mutations in a conserved ExxRxxR motif in the kinase domain (E519, R522, R525). This motif in other BY kinases is critical for oligomerization of their cytoplasmic domains [42–44]. The VpsO-503^E519A, R522A, R525A^ kinase domain can autophosphorylate its C-terminus similar to VpsO-503 (S1B Fig). We determined the structure of VpsO-503^E519A, R522A, R525A^ from x-ray diffraction data at a resolution of 2.9 Å (S1 Table). Two VpsO molecules are present in the asymmetric unit; these molecules dimerize through the α1 helix, which contains the mutated ExxRxxR motif (S1C Fig). The kinase domain adopts the expected α/β mononucleotide-binding fold with a seven-stranded β-sheet surrounded by eight α-helices (Fig 2B). The overall structure aligns closely with the structures of other BY-kinases; for example, the root mean square deviation of the 207 Cα atom distances between the VpsO and Wzc (PDB: 3LA6, chain A) kinase domains is 1.1 Å [43]. Comparison of the active sites revealed a similar arrangement of residues including those of the P-loop, which binds ATP (Fig 2B).

There are several notable differences in the VpsO pocket that accommodates adenosine relative to other BY-kinases. In Wzc, D480 in the N-terminal extension hydrogen bonds with the ribose [43]. In CapAB, the *Staphylococcus aureus* BY-kinase, residues from the CapA activating sequence (which corresponds to the N-terminal extension of Wzc) make van der Waals contacts with the adenine [42]. In contrast, the VpsO-503^E519A, R522A, R525A^ protein used for crystallization lacks the N-terminal extension found in other BY-kinases, and alternatively, its α1 helix is longer at its N-terminus. This helix likely contacts the adenine base of ATP, and it contains two glutamates (E512 and E516) that could coordinate the ribose (Fig 2B). The potential additional role of the α1 helix in VpsO binding ATP may explain why the N-terminal extension (residues 473–502) in VpsO is not required for catalytic activity (S1A Fig). Also, in contrast with other BY-kinases found in Gram-negative bacteria, VpsO lacks the tyrosine that coordinates the α-phosphate of ATP. In the *E*. *coli* BY-kinases Etk and Wzc, this tyrosine (Y569 in Wzc) blocks the active site until its phosphorylation results in an active conformation [45]. In VpsO, R583 occupies the equivalent position and may be involved in coordination of the α-phosphate (Fig 2B).

Refinement of the VpsO structure revealed unmodeled electron density in the ATP binding site that is consistent with the presence of a phosphorylated tyrosine residue (Fig 2C). The tyrosine-rich C-terminal tail is heterogeneously phosphorylated in the crystallized protein (S1B Fig). We did not observe electron density corresponding to the tail sequence elsewhere, suggesting it is primarily disordered except for the bound phosphotyrosine. The minimal density did not enable its assignment to a specific tyrosine, and considering the disorder in the tail, we propose that the density could be arising from more than one phosphorylated tyrosine in an equilibrium. The phosphate is located in a position similar to that of the β-phosphate of ADP in the Wzc structure [43]. It is coordinated through hydrogen bonding to backbone amides (residues 548–550) in the P-loop and through T552, which are all part of the canonical Walker A motif. In addition, R583 stacks against the tyrosine aromatic ring (Fig 2C). The position of the phosphotyrosine suggests that it should inhibit nucleotide binding, and we do not observe ADP in the active site despite its presence at 1 mM in the crystallization buffer. Thus, the structural data suggest that C-terminal tail phosphorylation results in a conformation that inhibits further catalytic activity.

### VpsU dephosphorylates VpsO

VpsU is a 166-amino-acid cytoplasmic protein containing the hallmark C(X)_5_R motif of the low-molecular-weight phosphotyrosine phosphatase (LMW-PTP) superfamily [30]. This motif is directly involved in the catalytic chemistry and the redox regulation of these proteins [30]. We purified VpsU and first analyzed its phosphotyrosine phosphatase activity using para-nitrophenylphosphate (pNPP) as a substrate. We also analyzed a mutated version of VpsU in which the residue cysteine 12, predicted to be critical for catalytic activity was replaced with a serine (VpsU^C12S^) to conserve protein structure [30,46]. Hydrolysis of pNPP was observed only for the reactions containing wild-type VpsU (Fig 2D). In a steady-state kinetic analysis of the wild-type activity, we measured the K_M_ to be 0.9 ± 0.1 mM and the k_cat_ to be 1.4 ± 0.1 min^-1^. These findings suggest that VpsU has phosphotyrosine phosphatase activity and that cysteine 12 is necessary for catalytic activity.

We next determined the crystal structure of VpsU at 2.2-Å resolution (S1 Table). The two molecules in the asymmetric unit form a domain-swapped dimer (Fig 2E). The overall fold of each monomer is similar to that of other prokaryotic and eukaryotic LMW-PTPs. It contains a central four-stranded parallel β-sheet flanked by two α-helices on one side and three α-helices on the other side. Comparison to the Wzb LMW-PTP from *E*. *coli* (PDB: 2WJA) revealed that the structures are similar (root mean square deviation of 105 Cα distance is 1.0 Å) [47]. VpsU contains the same complement of active site residues as other prokaryotic LMW-PTPs including the nucleophilic cysteine (C12) and the tyrosine (Y127) that stacks with the substrate phosphotyrosine. In the crystal, domain swapping in the dimer occurs through exchange of the C-terminal α-helix (α5). Although size-exclusion chromatography data were consistent with dimerization, we cannot be sure that domain swapping also occurs in solution.

To evaluate whether phosphorylated VpsO-503 is a substrate for VpsU, we assayed its phosphorylation state upon incubation with VpsU using a Phos-Tag gel. We found that in reactions containing VpsU, there was a complete shift of VpsO-503 to an unphosphorylated state (Fig 2F). The catalytically inactive VpsU^C12S^ mutant did not comparably dephosphorylate VpsO in this assay (S1D Fig). Moreover, VpsU had no effect on mobility when it was added to mutated versions of VpsO that were not capable of autophosphorylation (Fig 2F). Collectively, these results show that VpsU dephosphorylates VpsO *in vitro*.

### VpsO and VpsU contribute to VPS production

Having determined that VpsO is a tyrosine autokinase and that VpsU is a phosphotyrosine protein phosphatase, we next sought to determine how these enzymatic activities contribute to VPS production in *V*. *cholerae*. For these experiments we employed a rugose variant of a clinical *V*. *cholerae* O1 El Tor isolate (A1552) that produces ample matrix components and has readily screenable phenotypes, including corrugated colony morphology and biofilm formation, which are both dependent on VPS production and VPS interactions with matrix proteins [3,4,7,9,48].

**Fig 3:**
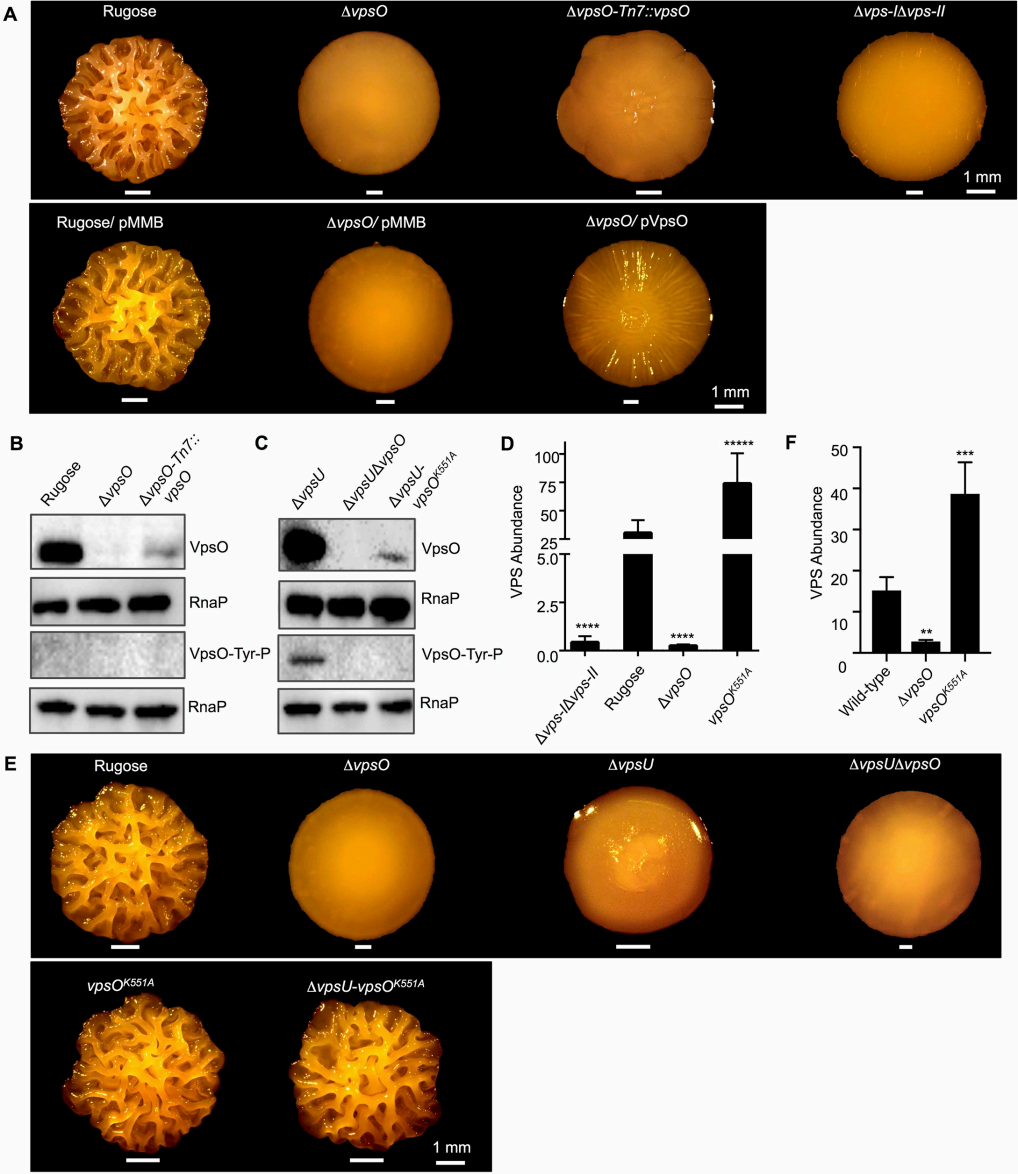
VpsO tyrosine kinase activity inhibits VPS production. **(A)** Colony corrugation phenotypes of complementation strains after 5 days of growth at 25°C. The expression of *vpsO* in the Δ*vpsO*-Tn7::*vpsO* strain is driven by the *lacI* promoter, and 1 mM IPTG is used for induction (top panel). The basal level of expression of *vpsO* in trans from pMMB in the Δ*vpsO/* pVpsO strain in the absence of induction, 0 mM IPTG (bottom panel) n≥3. **(B)** Western blot analysis for protein abundance and tyrosine phosphorylation of VpsO in the presence of 1 mM IPTG for *vpsO* induction by the *lacI* promoter; n≥3. **(C)** Western blot analysis for protein abundance and tyrosine phosphorylation of VpsO; n≥3. **(D)** VPS quantification in extracts of deletion and catalytically inactive mutant strains compared to the rugose strain; n = 2 for biological replicates each with n = 3 for technical replicates. ****, p < 0.001, *****, p < 0.0005 by one-way Anova multiple comparisons test. **(E)** Colony corrugation phenotypes after 5 days of growth at 25°C for the indicated strains; n≥3. **(F)** VPS quantification in extracts of deletion and catalytically inactive *vpsO* mutant strains in the wild-type background compared to wild-type; n = 2 for biological replicates each with n = 2 for technical replicates. **, p < 0.01, ***, p < 0.001 by one-way Anova multiple comparisons test.

Consistent with our earlier work, a *V*. *cholerae* rugose strain lacking *vpsO* lost colony corrugation and formed smooth colonies, similar in appearance to a strain harboring deletions of both *vps* clusters (Δ*vps-I*Δ*vps-II*, Fig 3A) [3,4]. This colony corrugation phenotype of the Δ*vpsO* strain was partially complemented by expression of VpsO from an IPTG-inducible promoter at a neutral Tn7 site on the chromosome or from a multicopy plasmid (Fig 3A). We surmise that partial complementation is most likely a function of mismatched stoichiometry of VPS biosynthesis assembly components since VpsO is normally expressed as part of a six-gene operon. We next analyzed the *in vivo* phosphorylation state of VpsO in extracts generated from exponentially grown cells, as *vpsO* expression is highest at this growth stage. Using a purified polyclonal antibody against VpsO, we observed that the protein was produced, but were unable to detect tyrosine phosphorylation via an anti-tyrosine antibody (Fig 3B). We reasoned that tyrosine phosphorylation was not detectable due to phosphotyrosine phosphatase activity in cellular extracts. Therefore, we analyzed the phosphorylation state of VpsO in strains lacking *vpsU*. In the absence of *vpsU*, we were able to readily observe tyrosine phosphorylation on VpsO (Fig 3C). In addition, we observed that VpsO protein abundance is increased in the Δ*vpsU* strain relative to the rugose strain, suggesting that VpsO tyrosine phosphorylation could increase protein stability.

To determine the *in vivo* consequences of VpsO phosphorylation on protein and tyrosine phosphorylation levels, we generated *V*. *cholerae* strains harboring the catalytically inactive K551A mutant of VpsO in both the rugose strain (*vpsO*^*K551A*^) and in the isogenic Δ*vpsU* strain (Δ*vpsU-vpsO*^*K551A*^). We found that in the Δ*vpsU-vpsO*^*K551A*^ strain, VpsO^K551A^ is produced at much lower abundance compared to VpsO in the Δ*vpsU* strain, and observed no tyrosine phosphorylation consistent with our *in vitro* results (Figs 2A and 3C and S1B Fig). We next analyzed VPS levels in these strains. While colony corrugation was similar, we found that VPS levels were enhanced in the *vpsO*^*K551A*^ strain compared to the rugose strain, and that VPS levels were abolished in the Δ*vpsO* strain (Fig 3D). Furthermore, colony corrugation of the *vpsO*^*K551A*^ and Δ*vpsU-vpsO*^*K551A*^ strains were similar (Fig 3E), as would be expected if VpsU is a cognate phosphatase to VpsO. To further confirm that the lack of VpsO tyrosine kinase activity enhances VPS production, we generated Δ*vpsO* and *vpsO*^*K551A*^ mutations in the wild-type *V*. *cholerae* O1 El Tor strain A1552 genetic background and quantified VPS production. VPS production was markedly higher in the *vpsO*^*K551A*^ strain when compared to the wild-type strain, while as expected VPS production was abrogated in the Δ*vpsO* strain (Fig 3F). Taken together, these findings indicate that VpsO tyrosine kinase activity inhibits VPS production.

**Fig 4:**
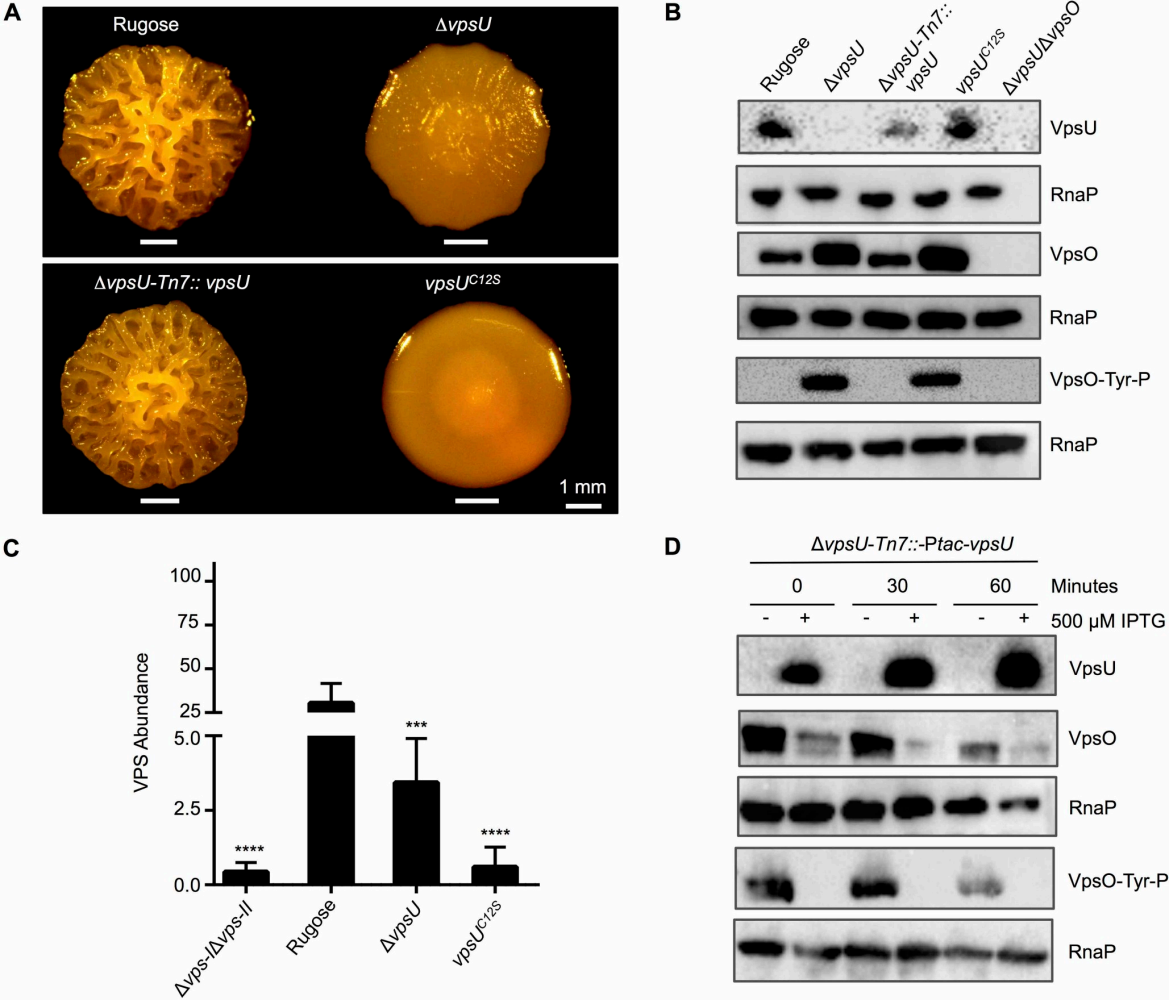
VpsU tyrosine phosphatase activity governs the VpsO tyrosine phosphorylation state and VpsO abundance. **(A)** Colony corrugation phenotypes after 5 days of growth at 25°C of the *vpsU* complementation strain under its native promoter and of the catalytically inactive mutant; n≥3. **(B)** Western blot analysis for VpsO abundance, tyrosine phosphorylation, and VpsU abundance in *vpsU* mutant backgrounds; n≥3. **(C)** VPS quantification in extracts of deletion and catalytically inactive mutant strains compared to the rugose strain; n = 2 for biological replicates each with n = 3 for technical replicates. ***, p < 0.005, ****, p < 0.001 by one-way Anova multiple comparisons test. The Δ*vps-I*Δ*vps-II* and rugose controls are identical to Fig 3D. **(D)** Western blot analysis for VpsO abundance, tyrosine phosphorylation, and VpsU abundance in the Δ*vpsU*-Tn7::P*tac*-*vpsU* strain, a *vpsU* inducible background in the presence of 100 μM chloramphenicol added at 0 minute; n≥2.

Given that VpsU can dephosphorylate VpsO (Fig 2F), we next sought to determine the impact of VpsU tyrosine phosphatase activity *in vivo*. Deletion of *vpsU* in the rugose variant (Δ*vpsU*), resulted in markedly reduced colony corrugation compared to the parental strain (Fig 4A). This phenotype was complemented by expression of *vpsU* from its native promoter at a neutral Tn7 site on the chromosome (Fig 4A). To assess the role of VpsU catalytic activity in VPS production, we generated a strain expressing catalytically inactive *vpsU*^*C12S*^. In contrast to Δ*vpsU*, the *vpsU*^*C12S*^ strain formed smoother colonies (Fig 4A). This observed phenotype is not due to altered protein production in the *vpsU*^*C12S*^ strain, as VpsU levels were similar in the parental strain, the *vpsU*^*C12S*^, and the Δ*vpsU*-Tn7::*vpsU* strain (Fig 4B). Consistent with the loss of colony corrugation, VPS production was abolished in the *vpsU*^*C12S*^ strain (Fig 4C). These results indicate that VpsU phosphotyrosine protein phosphatase activity stimulates VPS production. Additionally, as colony corrugation and VPS production were markedly reduced in the *vpsU*^*C12S*^ strain compared to the Δ*vpsU* strain, we surmise that VpsU might play additional non-catalytic roles in VPS production, perhaps through protein-protein interactions in the VPS biogenesis pathway.

As discussed above, in the strain lacking *vpsU* stability of VpsO is enhanced. To further investigate the impact of VpsU on VpsO stability, we next analyzed VpsO levels in the *vpsU* complementation strain and in the catalytically inactive strain. We found that either lack of VpsU or its phosphatase activity resulted in increased VpsO tyrosine phosphorylation, and increased VpsO abundance compared to the parental strain (Fig 4B). In addition, we performed an *in vivo* protein stability assay, following inhibition of translation by chloramphenicol, using a Δ*vpsU* strain in which VpsU production was placed under the control of the P*tac* promoter at the Tn7 locus on the chromosome. We observed that VpsO abundance and VpsO tyrosine phosphorylation decreased with increasing levels of VpsU (Fig 4D). Collectively, these results show that VpsU abundance and VpsU phosphotyrosine phosphatase activity together govern the phosphorylation state and abundance of VpsO.

### Tyrosine phosphorylation influences VpsO oligomerization and VPS production

To identify the VpsO residues that are phosphorylated *in vivo*, we expressed and purified an epitope-tagged version of VpsO (VpsO-Myc-His) in *V*. *cholerae*, and we identified the phosphorylated tyrosine residues using liquid chromatography mass spectrometry (LC-MS/MS). We identified phosphorylation of eight tyrosine residues (S2A Fig), five are located in the C-terminal tail of the kinase domain (Y717, Y720, Y721, Y726, and Y727), and the other three are located in the periplasm (Y72, Y150, and Y285). We also detected one phosphorylated serine (S718) residue (S2A Fig). S2 Table summarizes the phosphorylated peptides detected, and S2B and S2C Fig show representative spectra for a phosphorylated peptide from the periplasmic and from the cytoplasmic domain, respectively.

**Fig 5:**
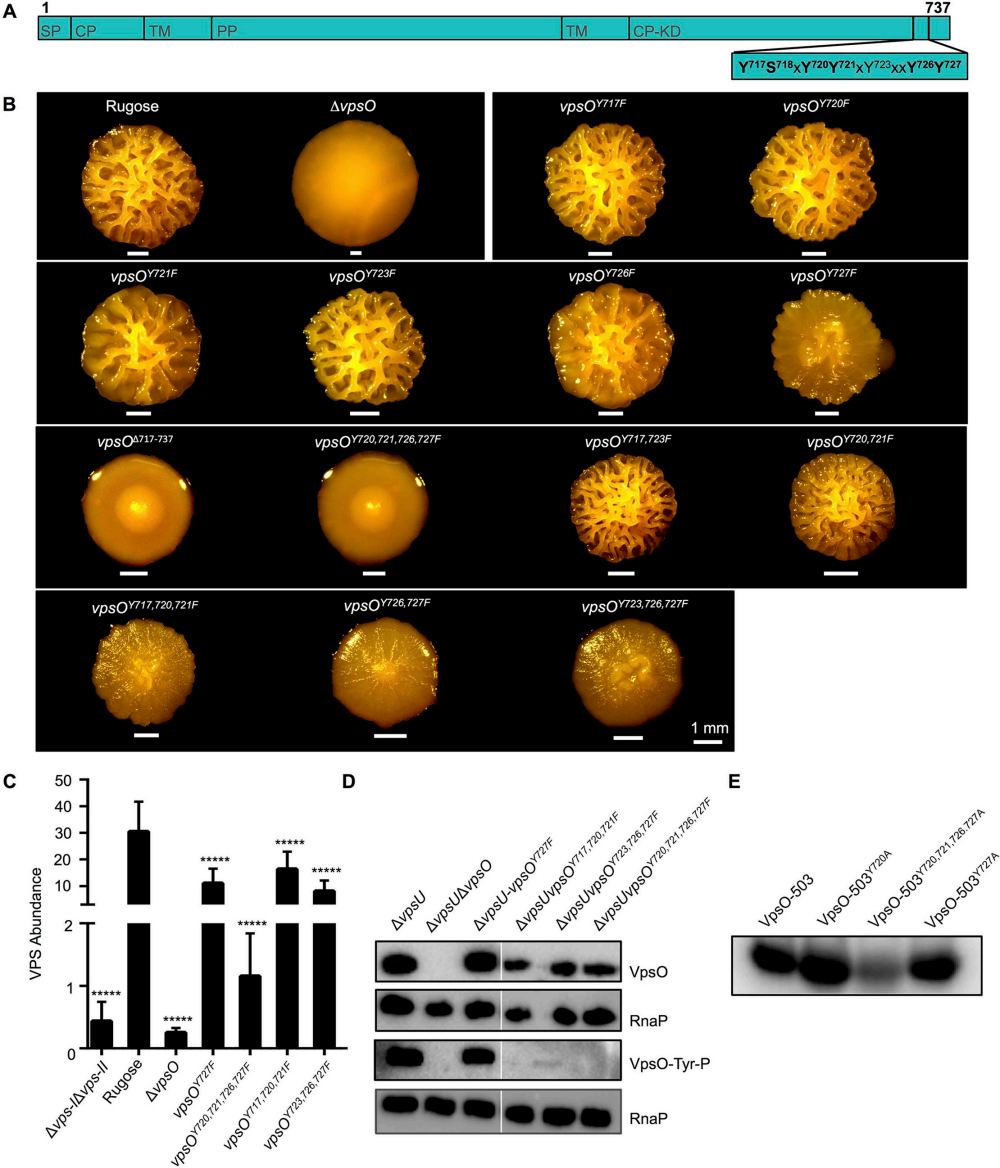
Phosphorylation of specific VpsO C-terminal tail tyrosine residues is critical for VpsO function. **(A**) Diagram of VpsO showing domain topology and the positions of the potential C-terminal tail phosphorylation sites. Signal peptide (SP), CytoPlasmic (CP), transmembrane (TM), Periplasmic (PP). CytoPlasmic Kinase Domain (CP-KD). **(B)** Colony corrugation phenotypes of the VpsO C-terminal tail mutants after 5 days of growth at 25°C. **(C)** Quantification of VPS in extracts of the C-terminal tail mutants compared to the rugose strain; n = 2 for biological replicates each with n = 3 for technical replicates. *****, p < 0.0005 by one-way Anova multiple comparisons test. **(D)** Western blot analysis for VpsO abundance and tyrosine phosphorylation; n≥3. **(E)** 50 μM VpsO-503 and the indicated three C-terminal tail mutants were incubated with [γ-^32^P]-ATP for 30 minutes and autophosphorylation was analyzed by gel electrophoresis and phosphorimaging; n≥3.

To investigate the importance of these tyrosines and their phosphorylation state, we first individually mutated all the C-terminal tail tyrosine residues (Y717, Y720, Y721, Y723, Y726, and Y727) to phenylalanine in the rugose strain (Fig 5A). A tyrosine to phenylalanine mutation makes the residue non-phosphorylatable, but we note it may also impact interactions made by the C-terminal tail [42,43]. We initially analyzed colony corrugation phenotypes of the single point-mutation strains, and only the *vpsO*^*Y727F*^ strain displayed a marked difference from the rugose strain, showing decreased colony corrugation (Fig 5B). Analysis of VPS production revealed that the *vpsO*^*Y727F*^ strain produced 3-fold less VPS compared to the rugose strain (Fig 5C).

We next generated strains harboring in-tandem combinations of point-mutations in the C-terminal tail (*vpsO*^*Δ717–737*^, *vpsO*^*Y720F*, *Y721F*, *Y726F*, *Y727F*^, *vpsO*^*Y720F*, *Y721F*^, *vpsO*^*Y717F*, *Y720F*, *Y721F*^, *vpsO*^*Y726F*, *Y727F*^, *vpsO*^*Y723F*, *Y726F*, *Y727F*^, *vpsO*^*Y717F*, *Y723F*^) (Fig 5A). We then analyzed colony corrugation phenotypes of the mutants (Fig 5B) and determined VPS production levels in strains exhibiting marked differences in colony corrugation (Fig 5C). Compared to the rugose strain, the *vpsO*^*Y717F*, *Y720F*, *Y721F*^ and *vpsO*^*Y723F*, *Y726F*, *Y727F*^ strains showed marked reduction in colony corrugation, as well as 2 and 4-fold decrease in VPS production, respectively (Fig 5B and 5C). However, the *vpsO*^*Y720F*, *Y721F*, *Y726F*, *Y727F*^ strain had a much greater reduction in colony corrugation, most similar to a strain lacking the C-terminal tail (*vpsO*^*Δ717–737*^) (Fig 5B). This significant reduction in corrugation correlated with 26-fold less VPS than the rugose parental strain (Fig 5B and 5C), though it was not as reduced as the Δ*vpsO* strain (117-fold less). These results suggest that mutations of the C-terminal tail phosphorylation sites do not phenocopy the effect of the K551A catalytically inactive kinase mutant. Instead phenotypes of C-terminal tail site mutations lead to a decrease in VPS levels.

We next sought to determine the impact of these tyrosine residues on VpsO abundance and tyrosine phosphorylation, by generating the single and in-tandem point mutations (Fig 5A and S3A Fig) in the Δ*vpsU* strain background. VpsO expression and tyrosine phosphorylation were similar in Δ*vpsU* and all the single point-mutant strains (Fig 5D and S3B Fig). Comparison of the Δ*vpsU* and the in-tandem point-mutation strains demonstrated a slight decrease in VpsO abundance accompanied by a marked decrease in tyrosine phosphorylation in all of the in-tandem point-mutation strains (Fig 5D and S3C Fig). To complement these *in vivo* tyrosine phosphorylation studies, we purified recombinant wild-type VpsO-503 and its mutated versions (VpsO-503^Y717A^, VpsO-503^Y720A^, VpsO-503^Y720A, Y721A, Y726A, Y727A^) and compared *in vitro* phosphorylation of these proteins. Consistent with *in vivo* tyrosine phosphorylation studies, decreased tyrosine phosphorylation was evident only in VpsO-503^Y720A, Y721A, Y726A, Y727A^ (Fig 5E). Collectively, these findings support our conclusion that autophosphorylation of the VpsO C-terminal tail increases VpsO stability, and that the position of tyrosine phosphorylation as well as number of phosphorylated tyrosine residues are critical for VPS production.

**Fig 6:**
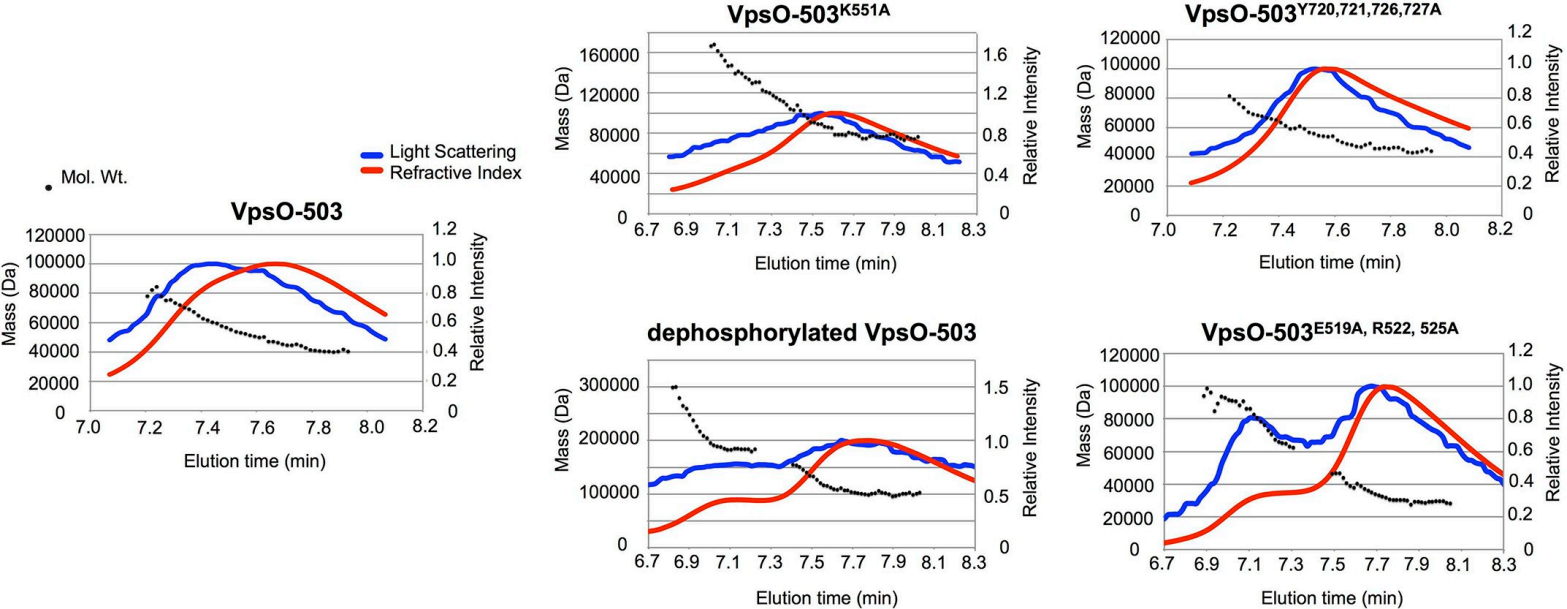
Tyrosine phosphorylation affects VpsO oligomerization. Multi-angle light scattering data for the indicated purified VpsO-503 kinase domain proteins. The peak(s) for each protein eluted from a size-exclusion chromatography column is shown with the relative intensity of light scattering (blue, right y-axis), refractometer signal (red, right y-axis), and the calculated molecular weight (black circles, left y-axis). Proteins were loaded at 1.5 mg/mL. A second peak, corresponding to a higher molecular weight species, could be detected in some experiments and likely reflects a concentration-dependent oligomerization equilibrium.

It has been observed that tail phosphorylation modulates the oligomerization state of other BY-kinases and that oligomerization impacts downstream signaling [42,43]. We next determined the impact of VpsO tail phosphorylation on VpsO oligomerization. We used multi-angle light scattering (MALS) to observe the oligomerization state of recombinant wild-type (VpsO-503), catalytically inactive (VpsO-503^K551A^), and VpsO with C-terminal tyrosine to phenylalanine mutations (VpsO-503^Y720A, Y721A, Y726A, Y727A^) (Fig 6). Although the variation in the molecular weight across the elution peak was too great to confidently determine the precise oligomerization state, we found that both the VpsO-503 and VpsO-503^Y720A, Y721A, Y726A, Y727A^ proteins had similar molecular weight profiles in the experiment (Fig 6). The molecular weight of the catalytically inactive VpsO-503^K551A^ was greater by up to 4-fold (Fig 6). Considering that the wild-type protein (VpsO-503) is heterogeneously phosphorylated after purification (S1B Fig), we also prepared dephosphorylated protein by mixing with VpsU prior to MALS. The dephosphorylated VpsO-503 had a higher molecular weight that was more similar to the catalytically inactive VpsO-503^K551A^. We conclude that phosphorylation of the C-terminal tail or tyrosine mutation disrupts oligomerization of the VpsO kinase domain (Fig 1B).

We note that in this *in vitro* oligomerization experiment VpsO-503 behaved similar to the “inactive” tail mutant, while *in vivo*, the wild-type VpsO strain maintained VPS production. We believe we observed this difference because the purified VpsO-503 is stably phosphorylated (S1B Fig) and behaves like the “inactive” low oligomerization state protein in the *in vitro* assay. In contrast, VpsO *in vivo* can cycle between a low and high oligomerization state, depending on its phosphorylation status. Thus, VpsO *in vivo* is more “active” compared to the VpsO-503 behavior *in vitro*.

### Analysis of the impact of tyrosine phosphorylation in the VpsO periplasmic domain

**Fig 7:**
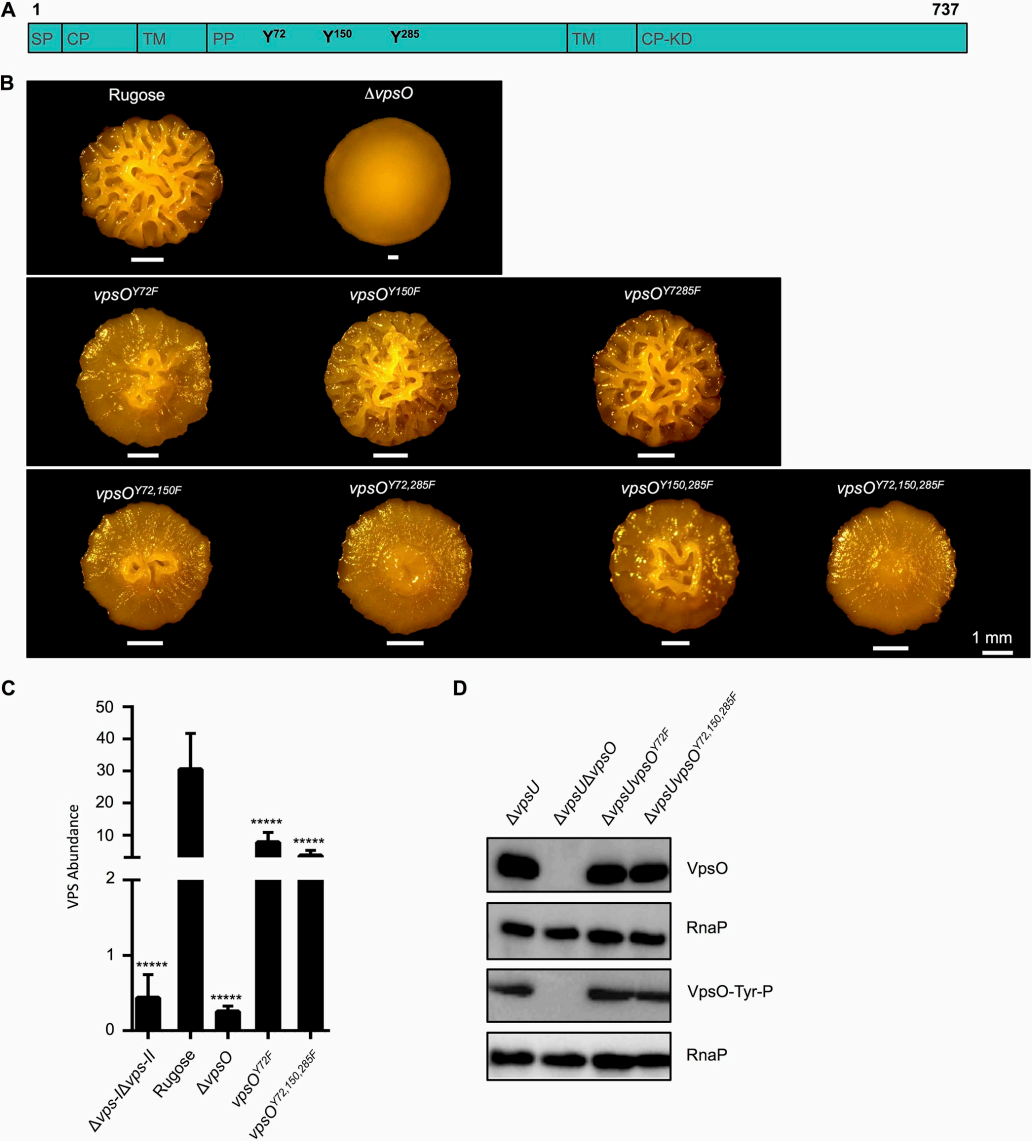
Phosphorylation of the periplasmic tyrosine residues impact VPS production without altering VpsO tyrosine phosphorylation. **(A)** Diagram of the VpsO protein highlighting the positions of the potential periplasmic domain phosphorylation sites. **(B)** Colony corrugation phenotypes of the periplasmic domain mutants after 5 days of growth at 25°C. **(C)** Quantification of VPS production by the periplasmic mutants compared to the rugose strain; n = 2 for biological replicates each with n = 3 for technical replicates. *****, p < 0.0005 by one-way Anova multiple comparisons test. The Δ*vps-I*Δ*vps-II*, rugose, Δ*vpsU*, and Δ*vpsO* control are identical to Fig 5C. **(D)** Western blot analysis of VpsO abundance and tyrosine phosphorylation; n≥3.

VpsO has a periplasmic domain (amino acid 45–448), and *in vivo* tyrosine phosphorylation analysis of VpsO revealed that the periplasmic domain of VpsO is phosphorylated at Y72, Y150, and Y285 (S2 Fig and S2 Table). To investigate the importance of tyrosine phosphorylation of these periplasmic tyrosine residues, we mutated them to phenylalanine singly and in-tandem, and we analyzed colony corrugation phenotypes and VPS production (Fig 7A). The VpsO^Y72F^ strain displayed markedly reduced colony corrugation (Fig 7B) and a 4-fold decrease in VPS production (Fig 7C). Strains harboring mutations in either two tyrosine residues (*vpsO*^*Y72F*, *Y150F*^, *vpsO*^*Y72F*, *Y285F*^, and *vpsO*^*Y150F*, *Y285F*^) or all three residues (*vpsO*^*Y72F*, *Y150F*, *Y285F*^) exhibited progressively greater decreases in colony corrugation (Fig 7B). Similarly, VPS production showed a 9-fold reduction in the *vpsO*^*Y72F*, *Y150F*, *Y285F*^ strain, and a 4-fold reduction in the *vpsO*^*Y72F*^ strain (Fig 7C).

We next analyzed the impact of the VpsO periplasmic domain tyrosine phosphorylation on overall VpsO protein stability and tyrosine phosphorylation in the Δ*vpsU* background. Neither VpsO abundance nor overall tyrosine phosphorylation was altered in these mutants compared to the Δ*vpsU* strain (Fig 7D and S4B Fig). Together, these results show that the number and position of phosphorylated tyrosine residues in the periplasmic domain are critical for stimulation of VPS production, without altering VpsO abundance or VpsO overall tyrosine phosphorylation potential.

### Analysis of the impact of the ExxRxxR motifs on VpsO phosphorylation and on VPS production

**Fig 8:**
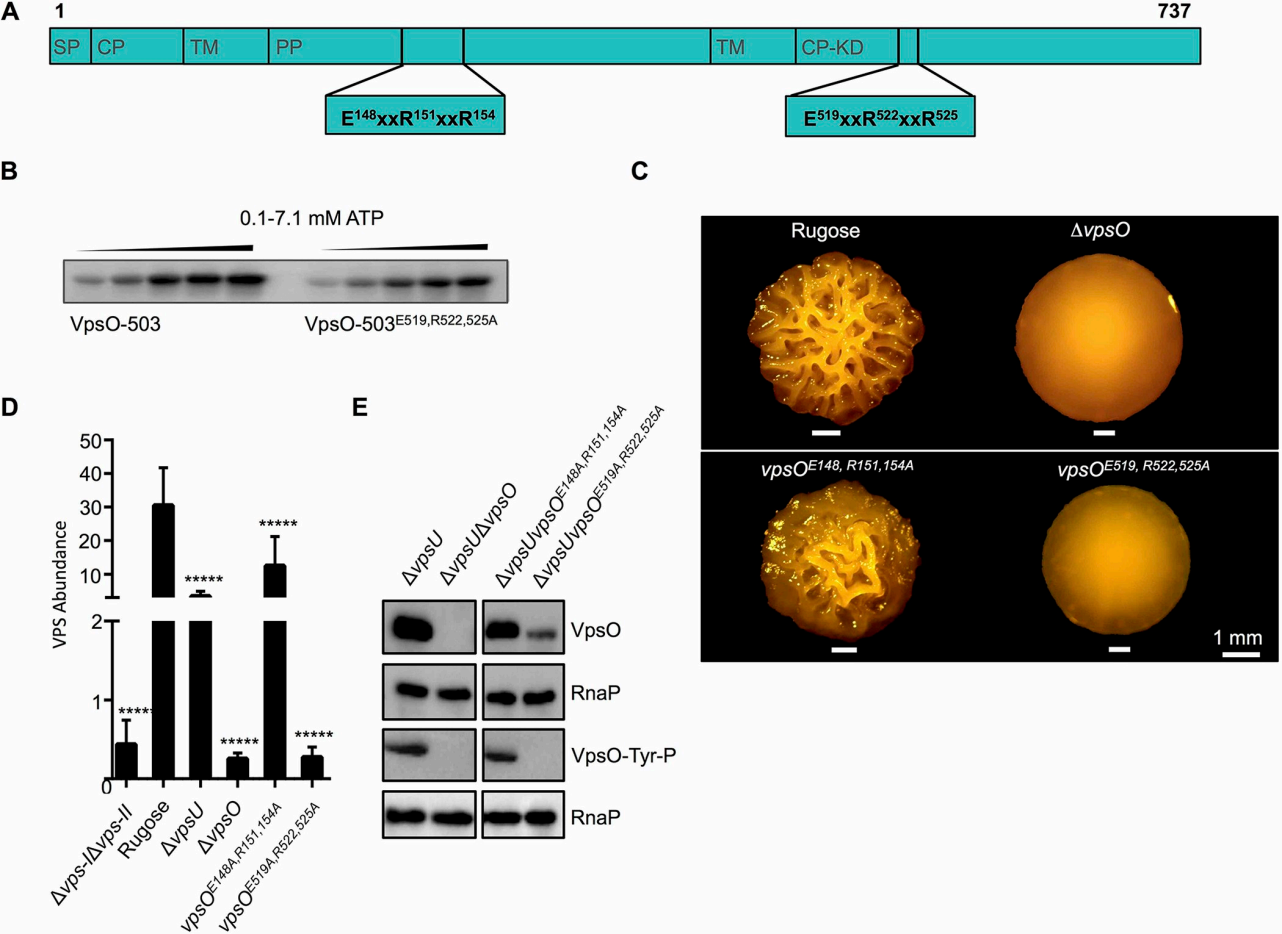
The ExxRxxR motifs alter VPS production *in vivo* and the VpsO oligomerization state without affecting kinase activity *in vitro*. **(A)** Diagram of the VpsO protein highlighting the positions of the ExxRxxR motifs. **(B)** 50 μM VpsO-503 and VpsO-503^E519A, R522A, R525A^ incubated with increasing concentrations of [γ-^32^P]-ATP were analyzed for autophosphorylation by gel electrophoresis; n≥2. **(C)** Colony corrugation phenotypes of the ExxRxxR motif mutants compared to the rugose strain after 5 days of growth at 25°C. **(D)** Quantification of VPS production by the periplasmic and ExxRxxR motif mutants compared to the rugose strain; n = 2 for biological replicates each with n = 3 for technical replicates. *****, p < 0.0005 by one-way Anova multiple comparisons test. The Δ*vps-I*Δ*vps-II*, rugose, Δ*vpsU*, and Δ*vpsO* control are identical to Fig 5C. **(E)** Western blot analysis of VpsO abundance and tyrosine phosphorylation; n≥3 The Δ*vpsU* and Δ*vpsU*Δ*vpsO* controls are identical to Fig 7D.

ExxRxxR motifs in BY-kinases facilitate oligomerization that is necessary for their function [42–44]. VpsO has two ExxRxxR motifs: one in the periplasmic domain (E148, R151, and R154) and one in the cytoplasmic kinase domain (E519, R522, and R525) (Fig 8A). MALS analysis confirmed that the VpsO-503^E519A, R522A, R525A^ domain, which has the mutated cytoplasmic ExxRxxR motif, has a similar molecular weight to the phosphorylated VpsO-503 (Fig 6), and in the crystal structure, VpsO-503^E519A, R522A, R525A^ appears to form a dimer (S1 Fig).

We compared tyrosine autophosphorylation of the purified VpsO-503 and VpsO-503^E519A, R522A, R525A^ kinase domains. We incubated the two proteins with increasing concentrations of [γ-^32^P]-ATP and observed the autokinase reactions under steady-state conditions. Both the wild-type VpsO-503 and mutant VpsO-503^E519A, R522A, R525A^ domains had similar activity profiles in this assay (Fig 8B), and we also found that VpsO-503^E519A, R522A, R525A^ was autophosphorylated to an extent similar to the wild type protein as assayed by electrospray mass spectrometry following purification (S1B Fig). These observations and results from other experiments, in which we analyzed autophosphorylation in mixtures of purified catalytically active and inactive VpsO-503 proteins, suggest that VpsO autophosphorylation occurs *in cis* (S7 Fig). Therefore, we conclude that the oligomeric state of the VpsO kinase domain does not impact *in vitro* autophosphorylation activity under the conditions of our assay.

We next sought to determine effects of these mutations *in vivo*. We generated strains with mutated versions of each ExxRxxR motif (*vpsO*^*E148A*, *R151A*, *R154A*^ and *vpsO*^*E519A*, *R522A*, *R525A*^) in the rugose strain. Mutation of the cytoplasmic ExxRxxR motif (*vpsO*^*E519A*, *R522A*, *R525A*^) resulted in colony corrugation and VPS levels similar to the Δ*vpsO* strain (Fig 8C and 8D), while mutation of the periplasmic motif (*vpsO*^*E148A*, *R151A*, *R154A*^) only showed slight decreases in colony corrugation and VPS production (2-fold) compared to the parental rugose strain (Fig 8C and 8D). Analysis of VpsO abundance and tyrosine phosphorylation, with periplasmic and cytoplasmic ExxRxxR motif mutants in the Δ*vpsU* background, demonstrated that only mutation of the cytoplasmic ExxRxxR motif (*vpsO*^*E519*, *R522*, *R525A*^) decreased overall VpsO abundance and attenuated observable VpsO phosphorylation (Fig 8E). VpsO abundance and tyrosine phosphorylation were unchanged in the periplasmic ExxRxxR motif mutant (*vpsO*^*E148*, *R151*, *R154A*^) (Fig 8E). These observations indicate that the cytoplasmic ExxRxxR motif is important for VpsO function. We propose that the cytoplasmic ExxRxxR motif stabilizes the oligomeric state of VpsO, which is necessary for VPS production (Fig 1B). The periplasmic ExxRxxR motif also contributes to VpsO function, albeit less than that of the cytoplasmic one, through a yet to be determined mechanism.

### Vps proteins are not phosphorylated in a VpsO dependent manner

BY-kinases can both catalyze autophosphorylation and phosphorylate other protein substrates *in trans*. Often, the targets of the tyrosine kinase involved in exopolysaccharide production are encoded by the exopolysaccharide clusters [28,29,39]. We therefore analyzed whether the proteins encoded within the VPS biosynthesis clusters are targets of VpsO. For these studies, we first expressed each of the Myc-His epitope tagged versions of the *vps* genes from an arabinose inducible promoter in respective deletion strains. We then analyzed production and tyrosine phosphorylation of each of the Vps proteins. We detected expression of all VPS cluster proteins except VpsI, and of these proteins only VpsO was observed to be tyrosine phosphorylated under the conditions tested (S5 Fig and S3 Table). These results suggest that VpsO solely autophosphorylates, or that additional targets are located outside of the VPS clusters.

### Analysis of the impact of tyrosine phosphorylation on biofilm structural properties and on biofilm fitness

**Fig 9:**
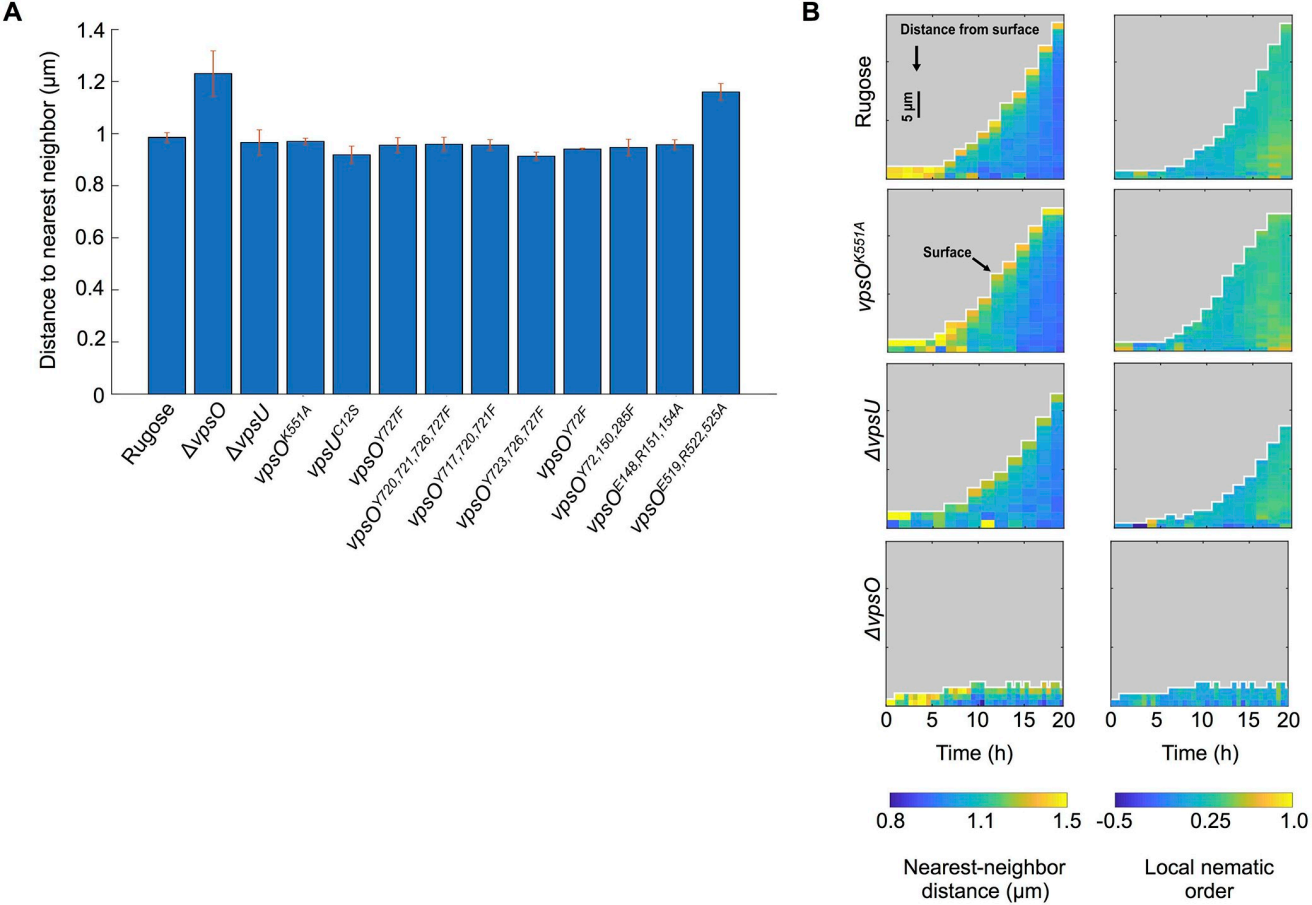
VPS influence biofilm architecture at single-cell level. **(A)** Nearest-neighbor distances in the context of distance (15 μm) from the center of biofilms under flow conditions for 21 hours; n = 3. **(B)** Nearest-neighbor distance and nematic order average analysis between cells in the context of time and height of flow cell biofilms of rugose, *vpsO*^*K551A*^, Δ*vpsU*, and Δ*vpsO*, n = 3.

To determine how tyrosine phosphorylation influences the development of biofilm architecture, we quantified key architectural parameters at the single cell level after 21 hours of biofilm growth under flow conditions. We measured the nearest-neighbor distance for cells that are located at a distance 15–20 μm from the center of the biofilm. This analysis was performed for the rugose, Δ*vpsO*, and Δ*vpsU* strains, for the catalytically inactive strains (*vpsO*^*K551A*^, *vpsU*^*C12S*^), for the strains harboring mutated versions of *vpsO* at various tyrosine residues, and for the strains with mutated ExxRxxR motifs (Fig 9A). The two non-VPS producing strains, Δ*vpsO* and *vpsO*^*E519A*, *R522A*, *R525A*^, had the highest nearest-neighbor distance, and they only formed a monolayer of cells on the glass surface, without 3D structure. The other mutants, which all formed 3D biofilm structures but displayed varying degrees of VPS production (Figs 3D, 3F, 4C, 5C, 7C and 8D), showed a similar nearest neighbor distance to the rugose strain.

To obtain further insights into the biofilm forming abilities of the Δ*vpsO*, Δ*vpsU*, and *vpsO*^*K551A*^ strains in comparison to the rugose strain, we additionally analyzed the biofilm development at single-cell resolution under flow conditions. This analysis resulted in measurements of the cell-cell spacing (quantified as the nearest-neighbor cell-to-cell distance) and the degree of cellular alignment in each cell’s local neighborhood (quantified as the local nematic order) (Fig 9B). All the strains have a higher nearest-neighbor cell-to-cell distance on the outer part of the biofilm compared to the center of the biofilm. By contrast, the local nematic order is highest at the center of the biofilm and decreases towards the outside of the biofilm, suggesting a correlation between cell density and cellular alignment. Qualitatively, the rugose, *vpsO*^*K551A*^, and Δ*vpsU* strains displayed similar spatiotemporal patterns for both the nearest-neighbor distance and the local nematic order. However, the Δ*vpsO* strain only developed a monolayer of cells attached to the surface with very small clusters, indicating that VPS is required for 3D biofilm formation. The Δ*vpsO* cell monolayers displayed lower nematic order and increased cell-cell spacing (Fig 9B) compared with the other strains. These results indicate that VPS is required for close cell-cell packing and 3D biofilm formation. However, very high levels of VPS (e.g. the *vpsO*^*K551A*^ strain) did not lead to tighter cell-cell packing under the biofilm growth conditions tested.

**Fig 10:**
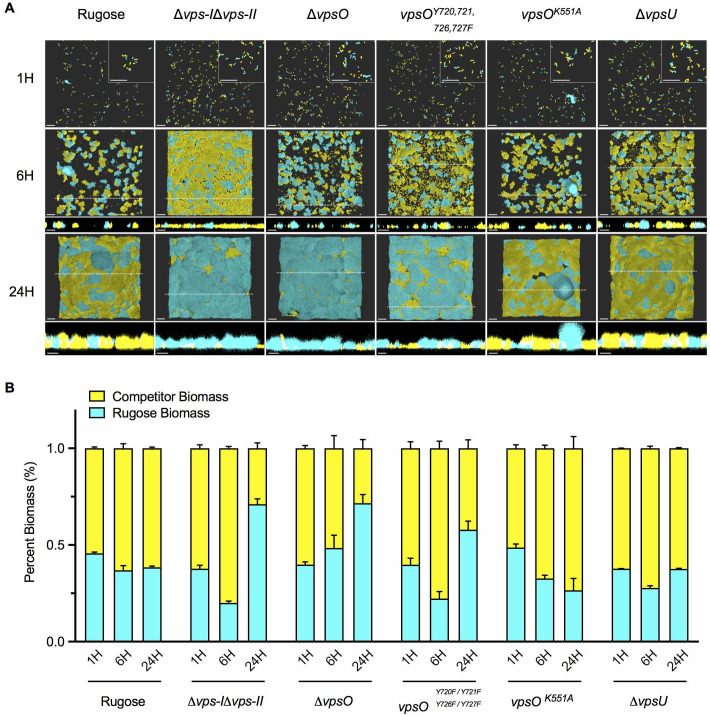
VPS production correlates with fitness in a flow cell model of biofilm competition. **(A)** Representative images of flow cell biofilm competition experiments between rugose::RFP (cyan) and mutant::GFP (yellow) obtained at 40x magnification at stages typical of initial surface attachment (1 hour, 1H), micro-colony development (6 hours, 6H), and mature biofilm formation (24 hours, 24H). Images were generated using Imaris software. Insets in the upper right corner of 1-hour images are zoomed in views of a region obtained from the same image to visualize single cells. Cross-sections of the XZ planes are shown below 6- and 24-hour images, and the dashed line on the image indicates the point from which the cross-section was obtained. Images are representative of two biological replicates, with three technical replicate images obtained per biological replicate. Scale bars = 20 μm. **(B)** Rugose (rugose::RFP, cyan) and competitor (mutant::GFP, yellow) biomass levels presented as a percentage of the overall biomass.

We next analyzed the contribution of VPS levels to biofilm fitness; we performed a biofilm competition assay using selected mutants with varying VPS production capabilities. This assay monitors the outcome of biofilm formation dynamics (micro-colony size and biomass) (S4 Table) when the rugose strain is competed against the Δ*vps-I*Δ*vps-II*, Δ*vpsO*, *vpsO*^*Y720F*, *Y721F*, *Y726F*, *Y727F*^, *vpsO*^*K551A*^, and Δ*vpsU* strains. When competed against the rugose strain, the Δ*vps-I*Δ*vps-II* strain initially had higher biomass but did not form micro-colonies (S4 Table). However, after 24 hours, a time point when the rugose strain forms a mature biofilm, the rugose strain outcompeted the Δ*vps-I*Δ*vps-II* strain (Fig 10A). Loss of *vpsO* led to deficiency in VPS production as it does in the Δ*vps-*Δ*vps-II* strain, but biofilm fitness of the Δ*vpsO* strain relative to the rugose strain drastically differed from the Δ*vps-I*Δ*vps-II* strain (Fig 10A). The biomass of the Δ*vpsO* strain was significantly reduced compared to the rugose strain at both 6- and 24-hours post biofilm initiation (51 and 28% of the total biomass, respectively) (Fig 10B). In addition, we observed that the Δ*vpsO* cells rounded only when grown in a flow cell, and that the biofilm fitness defect was partly due to detachment of these cells from the substratum (Fig 10A, S4 Table, S6 Fig and S1 Video). No rounded Δ*vpsO* cells were observed when cells were grown under planktonic or static biofilm conditions by either microscopy or scanning electron microscopy analyses of colonies (S6A and S6B Fig). This observation suggests that the production of rounded cells is induced only when cells are continuously supplied with fresh nutrients under flow conditions.

The *vpsO*^*K551A*^ strain had higher biomass than the parental rugose strain at 24 hours (*vpsO*^*K155A*^ was 74% of the total biomass; Fig 10B and S4 Table) consistent with the observed increase in VPS production. The strain harboring the mutated C-terminal tail tyrosine sites of VpsO (*vpsO*^*Y720F*, *Y721F*, *Y726F*, *Y727F*^) was markedly outcompeted by the rugose strain, but not as drastically as the Δ*vps-I*Δ*vps-II* strain (Fig 10A and 10B, and S4 Table). The Δ*vpsU* strain had decreased VPS production compared to the rugose strain, yet the Δ*vpsU* strain was not outcompeted by the rugose strain (Fig 10A and 10B), suggesting that the amount of VPS produced is sufficient for biofilm formation under the biofilm growth conditions tested. Collectively these studies show that the degree of VPS produced determines the dominance rank of the mutant strain when competed against rugose. The more VPS a strain produces, the better it competes for space in the biofilm competition model.

### Loss or disruption of VpsO oligomerization leads to vancomycin sensitivity

**Fig 11:**
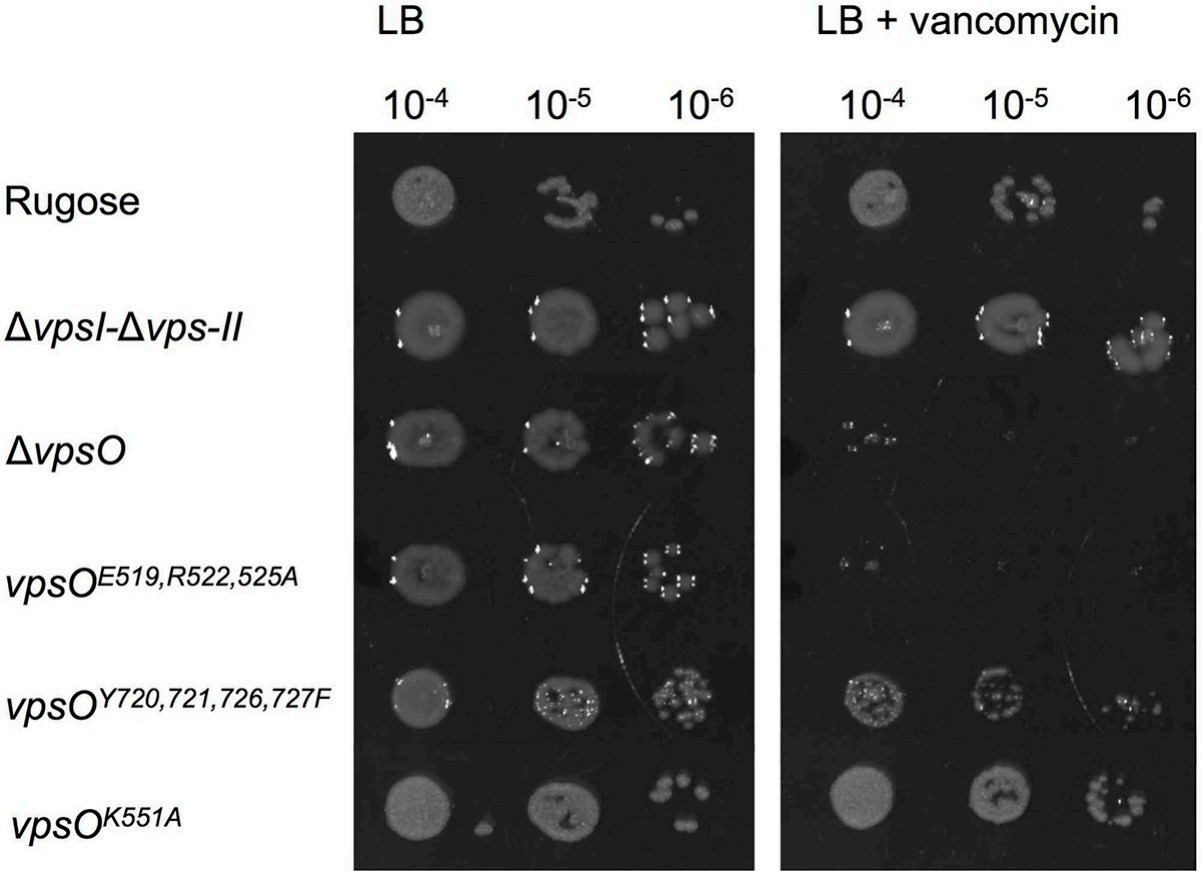
Lack of VpsO or loss of VpsO oligomerization renders rugose sensitive to vancomycin. Strains were plated onto LB agar and on LB agar containing vancomycin (200 μg/ml); n = 2 for biological replicates each with n = 2 for technical replicates.

Differences in biofilm formation and cell rounding phenotypes of Δ*vps-I*Δ*vps-II* and Δ*vpsO* strains prompted us to further investigate physiological consequences of these mutations (i.e. inability to produce VPS biosynthesis machinery completely as is the case for Δ*vps-I*Δ*vps-II* versus lacking only VpsO). It was reported that in the *V*. *cholerae* El Tor C6706 strain a transposon insertion in several of the *vps* genes rendered the cells sensitive to the antibiotic vancomycin [49]. We therefore investigated whether any of the strains harboring mutated versions of VpsO exhibited vancomycin sensitivity. Of Δ*vps-I*Δ*vps-II*, Δ*vpsO*, *vpsO*^*E519A*, *R522A*, *R525A*^, *vpsO*^*Y720F*, *Y721F*, *Y726F*, *Y727F*^, and *vpsO*^*K551A*^ strains, only Δ*vpsO* and *vpsO*^*E519A*, *R522A*, *R525A*^ were markedly sensitive to vancomycin and the *vpsO*^*Y720F*, *Y721F*, *Y726F*, *Y727F*^ strain exhibited increased vancomycin sensitivity compared to that of the rugose strain (Fig 11). In *E*. *coli* the BY-kinase Wzc and the outer membrane secretion pore Wza interact directly to form part of the polysaccharide biosynthesis complex, and BY-kinase homologs use the neighboring BY-kinase subunit’s tail for higher order oligomerization [42,50–53]. We reason that defects in VPS biosynthesis assembly, either the lack of or a decrease in the formation of VpsO higher order oligomers and/or the absence of high-molecular weight VPS, allows vancomycin to enter into the cells through the outer membrane polysaccharide export protein that normally secretes VPS. In the Δ*vps-I*Δ*vps-II* strain the predicted export protein would be absent, thus vancomycin cannot enter the cells, whereas in the case of VpsO^Y720F, Y721F, Y726F, Y727F^, VpsO oligomerizes, albeit to a lesser extend, as suggested by our *in vitro* MALS data (Fig 6) and based on homology through periplasmic domain interactions [53]. Therefore the partial assembly of the VPS machinery prevents entry of vancomycin into the cells leading to slight vancomycin sensitivity that we observed.

## Discussion

To understand the molecular requirements for building a robust biofilm matrix, we must identify the players and determine how they contribute to matrix production and the generation of a biofilm. Biofilm formation in *V*. *cholerae* requires the VPS polysaccharide, which is synthesized by *vps* gene products encoded in different operons. In this study, we explored the mechanisms involved in regulating biosynthesis of VPS, and we identified a tyrosine phosphoregulatory system (VpsO/VpsU) that post-translationally regulates VPS production. Bioinformatic analysis of *vps* gene products revealed that VPS is synthesized by a Wzy-dependent pathway similar to that of *Escherichia coli* group 1 capsular polysaccharide biosynthesis, which synthesizes and polymerizes exopolysaccharide repeat units and transfers the polymer across the outer membrane via multi-protein membrane complexes (Fig 1A) [4]. In the Wzy-dependent capsule biosynthesis pathway, the proteins Wza (outer membrane polysaccharide export protein), Wzc (tyrosine kinase), and Wzb (protein tyrosine phosphatase) are critical for capsule biosynthesis. Wza and Wzc are predicted to serve as molecular scaffolds that span the cell envelope to support assembly of the capsule biosynthesis/export machinery. It is also predicted that the tyrosine phosphorylation state of Wzc can modulate the protein-protein interactions required for assembly of the biosynthesis machinery [54].

The molecular mechansims by which Vps proteins govern VPS production, which protein(s) function as the outer membrane polysaccharide export protein, and which proteins interact to form the functional biosynthesis/export machinery remain poorly understood for the VPS production pathway. In this study, we explored the mechanisms involved in biosynthesis of VPS production by analyzing how the tyrosine phosphoregulatory system (VpsO/VpsU) post-translationally regulates VPS production. A strain lacking *vpsO* is unable to produce VPS, whereas a strain harboring a catalytically inactive kinase domain (*vpsO*^*K551A*^) is enhanced for VPS production (Fig 3D–3F). These observations support the hypothesis that VpsO is required for VPS biosynthesis but that its autophosphorylation inhibits VPS biosynthesis.

Tyrosine phosphorylation is reversible via the action of phosphotyrosine protein phosphatases, and here we observed that VpsU is a cognate protein tyrosine phosphatase for VpsO. A strain lacking *vpsU*, in which VpsO is produced and phosphorylated, demonstrated significantly decreased VPS production relative to the rugose strain (Fig 4B and 4C). We therefore think that cycling between high and low levels of C-terminal tyrosine-cluster phosphorylation states are important for VPS production, and our observations regarding VpsU further support the model that unphosphorylated VpsO is active toward VPS production.

We identified that tyrosine residues 717, 720, 721, 726, and 727 in the C-terminal tail of the VpsO kinase domain can be phosphorylated (S2 Fig). To analyze the importance of these residues, we mutated each tyrosine to a phenylalanine to inhibit its ability to be phosphorylated (Fig 5B). If phosphorylation inhibits VpsO functionality, we had predicted that C-terminal tail unphosphorylated mimics would show a phenotype similar to that of the catalytically inactive kinase mutant (*vpsO*^*K551A*^) with an increase in VPS production (Fig 3D–3F). However, for almost all the single and in-tandem mutants, we observed little change from WT or decreased VPS production and colony corrugation (Fig 5B and 5C). The decrease is particularly observable in strains with a mutation in Y727 (e.g. *vpsO*^*Y727F*^ and *vpsO*^*Y720F*, *Y721F*, *Y726F*, *Y727F*^). In fact, colony corrugation of the *vpsO*^*Y720F*, *Y721F*, *Y726F*, *Y727F*^ strain more closely resembled that of a C-terminal tail truncation (*vpsO*^*Δ717–737*^) than that of the *vpsO*^*K551A*^ strain. One possible explanation for these observations is that oligomerization of VpsO drives VPS production and that phosphorylation of the C-terminal tail or specific tyrosine mutations disrupt oligomerization (Fig 1B). If oligomerization is driven by interactions between tail tyrosines, as is the case with Wzc in *E*. *coli* and CapB in *S*. *aureus*, it is plausible that our tail deletion or tyrosine mutations also decrease oligomerization [42,43]. For example, in the structure of the unphosphorylated Wzc oligomer, Y715 makes an H bond using its hydroxyl to D564. This interaction is broken by either mutation to phenylalanine (losing OH group) or phosphorylation (phosphate minus charge and D564 minus charge destabilizing each other) [43]. We thus conclude that the tyrosine to phenylalanine mutation structurally and functionally mimics the phosphorylated state more than the unphosphorylated state (Fig 1B). Our *in vitro* MALS data with purified wild-type and catalytically inactive VpsO kinase domains are consistent with this model (Fig 6). The effect of *vpsU* deletion, namely increasing phosphorylation of VpsO and decreasing VPS production (Fig 4B and 4C), is also consistent with this model in that the phosphorylation state of VpsO determines its oligomerization state and functionality for VPS production. In contrast to the other tail tyrosine mutations, in-tandem tyrosine to phenylalanine mutation of residues 717 and 723 (*vpsO*^*Y717F*, *Y723F*^) did appear to enhance colony corrugation similar to the *vpsO*^*K551A*^ strain (Fig 5B). One possible explanation for this result is that these particular sites do not play a role in stabilizing the oligomer interface.

In other BY-kinases studied, ExxRxxR motifs found in the kinase domains are critical for oligomerization [23,42,44]. In *E*. *coli*, a mutation in the kinase domain ExxRxxR motif leads to a marked decrease in tyrosine phosphorylation and colanic acid production [43]. We observed a similar phenotype for the *V*. *cholerae* strain that expresses VpsO with a mutated ExxRxxR motif in the kinase domain (*vpsO*^*E519A*, *R522A*, *R525A*^), with a complete loss of VpsO tyrosine phosphorylation and VPS production (Fig 8C–8E). One difference we have observed in VpsO compared to other BY-kinases, is how oligomerization regulates autokinase activity. BY-kinases typically phosphorylate tyrosines *in trans* in C-terminal tails of neighboring kinases when they are assembled into oligomers *in vivo* [28,29,34,39]. A similar role for the ExxRxxR motif was observed for the BY-kinase Wzc in *E*. *coli* and CapB in *S*. *aureus* [42,43]. It was proposed that monomeric tyrosine phosphorylation occurs in solution as a result of transient interactions between subunits [42]. In the case of VpsO, our data indicate that autophosphorylation likely occurs *in cis* (Fig 8B and S7 Fig), which is consistent with the observation that the oligomerization state does not influence tail phosphorylation *in vitro*. It is possible that the difference in phosphorylation of *vpsO*^*E519A*, *R522A*, *R525A*^ *in vivo* and *in vitro* is a consequence of the absence of the periplasmic domain in the *in vitro* analyses (Fig 8B and 8E). In the full-length cryo-EM structure of the *E*. *coli* Wzc, the periplasmic domain is directly implicated in protein-protein interactions that aid in tetramer formation [53]. These periplasmic interactions may also be the reason why the C-terminal tail mutants (*vpsO*^*Y720F*, *Y721F*, *Y726F*, *Y727F*^ and *vpsO*^*Δ717–737*^) along with the Δ*vpsU* strain still retain low levels of VPS abundance (Figs 4C, 5B and 5C). However, the role of the periplasmic ExxRxxR motif in the function of BY-kinases is overall not well understood. This motif is conserved in the periplasmic domain of Wzc of *E*. *coli* and EpsA, the kinase modulator of EpsB of *Bacillus subtilis*, and VpsO; but their role has not been studied to date. In VpsO, the periplasmic ExxRxxR motif appears to contribute to overall VpsO function and VPS production, as mutation of these residues (*vpsO*^*E148A*, *R151A*, *R154A*^) results in decreased colony corrugation and VPS production (Fig 8C and 8D). However, the molecular mechanisms by which this takes place remain to be elucidated.

Several lines of evidence from our data also suggest that the phosphorylation state of VpsO may regulate its proteolytic degradation. Loss of tyrosine autophosphorylation in the catalytically inactive *vpsO*^*K551A*^ strain resulted in a decrease in total VpsO protein abundance compared to the rugose strain (Fig 3C). Conversely, loss of phosphatase activity (Δ*vpsU* or *vpsU*^*C12S*^) and the resulting increase in VpsO tyrosine phosphorylation resulted in an increase in total VpsO protein levels (Fig 4B). In the Gram-positive bacterium *B*. *subtilis*, proteolytic degradation, instead of dephosphorylation via a phosphatase, controls the tyrosine phosphorylation state of the BY-kinase EpsB [32]. The exopolysaccharide phosphoregulatory system in *V*. *cholerae* functions differently from that of *B*. *subtilis*, since VpsU actively dephosphorylates VpsO, yet proteolytic degradation also appears to play a regulatory role. Perhaps enhanced proteolytic degradation of unphosphorylated VpsO serves as a secondary regulatory mechanism to control VpsO functionality and VPS production in response to a yet unknown signal, despite the presence and/or activity of the VpsU phosphatase. Therefore, our results suggest that the *V*. *cholerae* tyrosine kinase VpsO is unique in that it employs both dephosphorylation and proteolytic degradation to regulate VPS production.

Regulation of the VPS biosynthesis pathway by these multiple post-translational mechanisms illustrates the importance of tight regulation of VPS production for efficient biofilm formation and coordination with other biofilm components, including biofilm matrix proteins. In yet another potential layer of regulation, our biochemical and structural observations suggest that VpsO autophosphorylation inhibits its kinase activity: first, electron density in the VpsO crystal structure appears in the active site ATP binding-pocket and likely marks the presence of an inhibitory phosphotyrosine (Fig 2C). Second, while we observed autocatalytic activity of VpsO, we did not observe this autokinase activity *in trans* when we assayed VpsO phosphorylation of a catalytically inactive VpsO fusion protein (GST-VpsO-503^K551A^) (S7A Fig). Together these data suggest a model in which autophosphorylation of the C-terminal tail occurs rapidly and subsequently the tail binds in the active site. One explanation is that some of the tail phosphorylation sites are especially important for oligomerization [for example Y727 as suggested by our data (Fig 5B)], while others are more important for regulating phosphorylation of the “oligomerization sites” through the autoinhibition mechanism. In this way, the specific pattern of phosphorylation as well as the overall fluctuation in level of phosphorylation may provide for fine-tuning of the signaling toward VPS production. Additional studies are needed to further test this model.

We evaluated the impact of tyrosine phosphorylation on biofilm structural properties and in biofilm fitness. Single-cell resolution analysis of biofilm formation showed that VPS production is required for native cell-cell packing and 3D biofilm organization (Fig 9). Biofilm competition experiments demonstrated that strains lacking VPS are not impaired for initial surface attachment and colonization, yet they are unable to develop typical three-dimensional micro-colonies. As these strains do not experience the high energetic costs of VPS production, they did have enhanced growth fitness, and produced more biomass than the rugose strain early after initial attachment (Fig 10). Temporal analysis of biofilm formation revealed that biofilm properties such as biomass, both total number and average volume of microcolonies at the substratum, are different between Δ*vpsU* and *vpsO*^*K551A*^ (S4 Table). We note that the strain lacking VpsU, which has reduced VPS levels, did not exhibit any biofilm formation defects relative to the rugose strain under the conditions tested. Together, these results highlight the importance of native levels of VPS production and the timing of production for the formation of *V*. *cholerae* biofilms.

The Δ*vpsO* strain was an exception in the biofilm competition experiment, as this strain had lower fitness and lower biomass levels over the time course than did the parental strain, despite the lack of VPS. We observed cell rounding (not associated with cell death) and increased detachment of the Δ*vpsO* strain under flow, nutrient-rich and high cell division conditions (Fig 10 and S6 Fig). Cell rounding is typically associated with modulations in the peptidoglycan layer, which is in high flux under increased growth conditions and tightly associated with the cell division and rod maintenance machinery [55]. We hypothesize that as in *Streptococcus pneumonia* [56], the exopolysaccharide production machinery may also be linked to cell division in *V*. *cholerae*; further studies are necessary to elucidate a potential orchestrated interplay.

Our findings additionally suggest that proteins involved in VPS production could serve as novel drug targets to inhibit biofilm formation in *V*. *cholerae* (Fig 11). The development of BY-kinase inhibitors has been pursued as a promising strategy to combat biofilm infections due to differences in the mechanisms of action between eukaryotic and prokaryotic tyrosine kinases.

## Materials and methods

### Bacterial strains and culture conditions

The strains and plasmids used in this study are listed in S5 Table. Mutations were generated predominantly in the rugose strain of *V*. *cholerae*, except for the data in Fig 3E, which was in the wild-type strain. *V*. *cholerae* and *E*. *coli* strains were grown aerobically in Luria-Bertani (LB) broth (1% tryptone, 0.5% yeast extract, 1% NaCl, [pH 7.5]) at 30°C and 37°C, respectively. Granulated agar (Difco) at 1.5% (wt/ vol) was used for LB agar medium. Concentrations of medium additives when necessary were as follows: 100 μg/μl rifampicin; 100 μg/μl ampicillin; 30 μg/mL gentamicin, 1 mM isopropyl b-D-1-thiogalactopyranoside (IPTG); and 0.05% arabinose.

### Strain and plasmid construction

Standard cloning methods or the Gibson assembly recombinant DNA technique (New England BioLabs) were used for plasmid construction. Polymerase chain reactions (PCR) were carried out with primers purchased from Integrated DNA Technologies and Q5 High-Fidelity master mix (New England BioLabs). Sequencing, performed at the UC Berkeley DNA Sequencing Facility, was used for construct verification. All *V*. *cholerae* sequences were amplified from genomic DNA isolated from *V*. *cholerae* A1552 strain. For chromosomal point mutant constructs, the native gene, including 500 base pairs (bp) upstream and downstream, was cloned into the pGP704sacB plasmid. This construct was then used for mutagenesis with the Q5 mutagenesis kit (New England BioLabs) to create the point mutants. Alternatively, for chromosomal point mutant constructs, the native gene, carrying the point mutation including 500 bp upstream and downstream, was cloned into the pGP704sacB plasmid. Gene replacements were then carried out via allelic exchange in the deletion background strain as previously described [3]. Complementation of both *vpsO* and *vpsU* was carried out using the Tn7-based system described previously [57]. The open reading frame (ORF) of *vpsO* under control of the *lac* promoter, which was amplified from pMAL-c5X, and the ORF of *vpsU* under control of its native promoter, which involves 319 bp of genomic sequence upstream of the transcription start site, was placed in the conserved Tn7 site at the 3’ end of the *glmS* gene. Expression constructs pBAD-VpsO-Myc/His and p2GT-VpsU were cloned by Berkeley MacroLabs. The expression construct p2GT-*vpsO*^*473*^ was constructed via Gibson assembly starting at residue 473 with an N-terminal TEV cleavable GST-tag. pGEV-*vpsO*^*503*^ was cloned between EcoRI and XhoI sites starting at residue 503 with an N-terminal TEV cleavable GST-tag. Mutated versions of pGEV-vpsO^503^ were constructed using Q5 mutagenesis. The complementation construct pMMB-*vpsO* was constructed via Gibson assembly.

### Recombinant protein expression and purification

VpsO and VpsU constructs were expressed as GST-fusion proteins from a pGEX or p2GT vector, respectively, in *E*. *coli* BL21(DE3). Transformed cells were grown to an OD_600_ between 0.6 and 1, and expression was induced with 1 mM IPTG overnight at 20°C. Cell pellets were resuspended in a lysis buffer containing 25 mM Tris (pH 8.0), 200 mM sodium chloride, 1 mM dithiothreitol (DTT), and 1 mM phenylmethylsulfonyl fluoride (PMSF). Cells were lysed with a homogenizer (C3 Emulsiflex, Avestin), and the supernatant was passed over a glutathione sepharose affinity chromatography column equilibrated with lysis buffer. The GST-fusion protein was eluted in lysis buffer containing 20 mM glutathione. The fusion protein was then further purified using anion exchange chromatography and cleaved using TEV protease overnight at 4°C. The target protein was separated from GST by repassing over glutathione sepharose. The protein was prepared for crystallization or biochemical assays by final purification using a HiLoad Superdex 200 (GE Healthcare) column equilibrated in a buffer containing 25 mM Tris (pH 8.0), 50 mM NaCl, and 1 mM DTT.

Wild-type VpsO-503 and VpsO-503^E519A, R522A, R525A^ were found to be heterogeneously phosphorylated following purification, while the VpsO-503^K551A^ mutant showed no phosphorylation (S1B Fig). To prepare dephosphorylated protein for enzymatic assays, VpsO was mixed with an equal mass of GST-VpsU and incubated at 37°C for 1 hour in a buffer containing 40 mM Tris (pH 8), 150 mM NaCl, and 10 mM DTT. The GST-VpsU protein was then removed using glutathione affinity capture and the Superdex 200 column.

### Crystallization and structure determination

Crystals of the VpsO-503^E519A, R522A, R525A^ kinase domain were grown using the hanging drop method at 20°C. Crystals were obtained by mixing 1 μl of protein (18 mg/ml, 25 mM Tris [pH 8.0], 50 mM NaCl, and 1 mM DTT) with an equal amount of crystallization solution (0.2 M sodium thiocyanate [pH 6.9], and 20% PEG 3350). Crystals were harvested and flash frozen in the crystallization solution with 20% glycerol before data collection. Purified VpsU was crystallized following concentration to 15 mg/ml. Crystals were grown by sitting-drop vapor diffusion at 20°C in a solution containing 0.1 M HEPES (pH 7.5), 1.25 M tri-sodium citrate, and 10% glycerol. Crystals were harvested and flash frozen in the same solution with 20% glycerol.

Data were collected from a single crystal at λ = 1.0 Å, 100 K on Beamline 8.3.1 at the Advanced Light Source, Lawrence Berkeley National Laboratory and Beamline 23-ID-B at the Advanced Photon Source, Argonne National Laboratory. Diffraction images were indexed and scaled using Mosflm and Scala in the CCP4 package [58]. Phases were solved by molecular replacement with PHASER [59]. The crystal structure of the chimeric protein CapA1B1 (PDB: 4JLV) was used as a search model for VpsO, and the crystal structure of the low molecular weight phosphotyrosine phosphatase VC0395 (PDB: 4LRQ) was used as a search model for VpsU. The resulting models were refined with Phenix [59,60]. NCS restraints were used throughout the refinement, and the inclusion of TLS refinement was implemented in the latter stages of refinement. All reflections were used for refinement except for 5% were excluded for R_free_ calculations. The structural model was revised in real space with the program COOT based on sigma-A weighted 2Fo-Fc and Fo-Fc electron density maps. Data collection and final refinement statistics are given in S1 Table. The VpsO-503^E519A, R522A, R525A^ and VpsU structures are available in the Protein Data Bank with accession codes 6U1Q and 6U1P, respectively.

### Enzyme assays

VpsO kinase reactions were performed in a volume of 30 μL in a buffer containing 25 mM Hepes (pH 7.0), 200 mM NaCl, 10 mM MgCl_2_, 1 mM DTT, and 100 μM (radiolabeling assay) or 400 μM ATP (Phos-Tag assay). Autokinase activity was assayed using 10 or 50 μM VpsO and 100 μCi of [γ-^32^P]-ATP where indicated. Kinase reactions analyzed by Phos-Tag gels were performed at 37°C, while reactions with [γ-^32^P]-ATP were performed at room temperature. In the Phos-Tag experiment shown in Fig 2A, the WT and tail mutant VpsO-503 proteins were prepared in a dephosphorylated state as described above prior to the kinase reaction. Phos-Tag gel electrophoresis in Fig 2A and 2F was performed using homemade SDS-PAGE gels incorporating Phos-Tag-acrylamide (Wako). The experiment in S1D Fig was performed using pre-cast SuperSep Phos-Tag gels (Wako). Phos-Tag gels were stained with Coomassie blue. Phosphorimaging was performed with a Typhoon imager (GE Healthcare Life Sciences). Electrospray mass spectrometry was performed using a Sciex X500B QTOF system, and the reaction (containing ~1 μg VpsO) was injected onto an in-line C-18 reverse-phase column.

Colorimetric phosphatase assays of VpsU activity were performed using p-nitrophenyl phosphate (pNPP). Product formation was detected by absorption of 405 nm light. Activity from 1 μM enzyme was assayed during a 3-hour reaction at the indicated concentration of pNPP at 37°C in 40 mM Tris (pH 8), 150 mM NaCl, 10 mM DTT, and 1% Tween. We found that the reducing agent is critical for VpsU activity. To measure dephosphorylation of VpsO, 10 μM VpsU was added to 10 μM VpsO and incubated for 30 minutes at 37°C.

### Multi-Angle Light Scattering

Molecular weights were obtained by static light scattering using a Wyatt Optilab T-rEX refractometer and miniDAWN Treos multiangle light scattering system at room temperature. The MALS detection system was in line with a size-exclusion chromatography (SEC) system driven by an HPLC pump with manual injector (Agilent Technologies). 25 μL of each VpsO protein at a concentration of 1.5 mg/mL was injected onto a silica-based SEC analytical HPLC column (5 μm, 500 Angstrom, 4.6 mm ID; Wyatt Technology), equilibrated in a running buffer containing 25 mM Tris (pH 8.0), 50 mM NaCl, and 1 mM DTT. Protein concentration was monitored by a refractometer and light scattering directly after elution from the SEC column. Absolute molecular weights were determined using ASTRA version 6.0 analysis software (Wyatt Technologies).

### Western blot analyses

A 1/200 dilution from an overnight culture of *V*. *cholerae* was inoculated into 125 ml flasks containing 50 ml of LB. For *vpsO* complementation assays, the cultures were grown for 40 minutes and then induced with 1 mM IPTG for 2 hours. For analysis of VpsO stability following translational inhibition, the cultures were grown to an OD_600_ of between 0.8 and 0.9 and induced with 500 μM IPTG for 2 hours, after which 100 μM chloramphenicol was added and 5 ml samples were harvested after 30 and 60 minutes. Samples were treated as indicated below, but pellets were only resuspended in 200 μl sterile MilliQ water. For all other western blot experiments: The cultures were grown to an OD_600_ of between 0.3 and 0.4. Cells were pelted at 4000 RPM for 20 minutes at 4°C. After the supernatant was decanted, the pellets were kept on ice and resuspended in 2 ml sterile MilliQ water containing the following protease and phosphatase inhibitors (if blotting for tyrosine phosphorylation): SigmaFast EDTA-free protease inhibitor cocktail, 10 mM NaF, and phosphatase inhibitor cocktails 2 & 3 (Sigma-Aldrich). Next, cells were lysed and proteins denatured by adding 10% sodium dodecyl sulfate (Sigma-Aldrich) to a final concentration of 2%. The denatured cell lysates were then heated for 15 minutes at 91°C.

Protein concentrations were determined using a Pierce BCA protein assay kit (Thermo Fisher). For VpsO and VpsU protein abundance 30 μg of protein was loaded, for VpsO-Tyr-P 80 μg of protein was loaded. RNA polymerase was used as loading control. In addition, we identified a protein which was recognized by the tyrosine phosphorylation antibody, whose abundance does not change in the samples used. This protein is used as loading control too in the supplemental figures. After SDS-PAGE electrophoresis the proteins were transferred onto a PVDF membrane (Immobilon, 0.45 μm, Millipore) and probed via immunoblotting. The following antibodies were used for immunoblotting: anti-VpsO generated against the kinase domain (Cocalico; 1:1000); anti-VpsU (GenScript; 1:1000); anti-phosphorylated tyrosine (Millipore; 1:1000); anti-RnaP (Biolegend; 1:2500–1:5000); mouse anti-rabbit horseradish peroxidase-conjugated (Santa Cruz Biotech, 1:1000), and goat anti-mouse horseradish peroxidase-conjugated (Invitrogen; 1:2500–1:5000). The immunoblots were developed with the SuperSignal West Pico chemiluminescent kit (Pierce). Immunoblot analyses were carried out with minimum two biological replicates.

### VPS purification and quantification

VPS purification was adapted from a previously published protocol [9]. Overnight cultures grown in LB were plated on LB agar plates covered with sterile dialysis membrane (Fisher Scientific) and grown overnight at 30°C. Upon harvest, the cells were resuspended in 10 mM Tris (pH 8.0). Normalization was achieved by adjusting each sample to the same OD_600_. Equal volumes were pipetted into 2-ml Eppendorf tubes and rotated for 1 hour at 4°C. To separate the matrix material from the cells, the suspension was centrifuged twice at 15000 x g for 30 minutes at 4°C. The supernatant fraction was recovered each time and after the second centrifugation step precipitated in 3 volumes of ice-cold ethanol at -20°C overnight. Crude VPS was then pelleted at 21130 x g for 30 minutes at 4°C followed by a 70% ethanol wash. The VPS pellets were then dried on ice and resuspended in 200 μl sterile MilliQ water.

For quantification, the VPS was serially diluted and 3 μl of each dilution was spotted on nitrocellulose membrane (Protran, 0.2 μm), followed by immunoblot analysis with anti-VPS antiserum (1:1000) and goat anti-rabbit horseradishperoxidase-conjugated antibody (1:2500). The VPS antibody used here is polyclonal and with undefined epitopes. The immunoblots were developed with the SuperSignal West Pico chemiluminescent kit (Pierce) and quantified using Fiji imageJ software (NIH). VPS immunoblot analyses were carried out with two different biological replicates and three technical replicates.

### Analysis of colony corrugation

For morphology imaging, 20 ml of LB agar was added per plate, and the plates were dried at room temperature for one day before use. For VpsO complementation, the agar contained 1 mM IPTG, for all other strains LB agar only was used. For colony morphology, the overnight cultures were diluted to 10^−8^ and grown at 25°C for 5 days. Colony morphology was imaged with the Zeiss Stemi 2000-C microscope equipped with Zeiss AxioCam ERc 5 s Microscope Camera. Morphology experiments were carried out with minimum two biological replicates.

### VpsO purification and determination of tyrosine phosphorylation

The Δ*vpsO*Δ*ctxAB* pBAD B *vps*O-Myc/His strain was used to purify full-length VpsO-Myc/His from *V*. *cholerae* to determine its native phosphorylation sites. The purification protocol was adapted from a previously published method [53]. Three liters of *V*. *cholerae* culture was grown to an OD_600_ of between 0.3 and 0.4 and induced with 0.05% arabinose for 3 hours. Next, the cells were pelleted (8200 x g for 15 minutes at 4°C), resuspended in lysis buffer (20 mM sodium phosphate [pH 7.0], 1 mM PMSF, 50 mM sodium fluoride, 100 mM β-glycerol phosphate), and lysed using a homogenizer (C3 Emulsiflex, Avestin). The clarified lysate (two centrifugation spins: 20200 x g for 30 minutes at 4°C) was then ultra-centrifuged for 1 hour at 34000 RPM. The membrane fraction was then resuspended in solubilization buffer (20 mM sodium phosphate [pH 7.0], 500 mM NaCl, 0.1% dodecyl maltoside [DDM], 50 mM sodium fluoride, 100 mM β-glycerol phosphate) and incubated rocking overnight at 4°C. The soluble fraction was then collected after ultra-centrifuging at 85000 x g for 1 hour at 4°C and incubated with nickel resin overnight. The wash buffer used for this purification was the same as the solubilization buffer but with reduced DDM (0.008%) and 10 mM imidazole added. The elution buffer used was the same as the wash buffer but with 200 mM imidazole. After the protein was concentrated, it was dialyzed into 20 mM Tris (pH 7.0), 300 mM NaCl, 0.008% DDM. The sample was then analyzed via SDS-PAGE.

## Preparation of samples for mass spectrometry

The gel band containing VpsO was excised, cut into 1-mm^3^ pieces and destained for 15 minutes in a 1:1 (v/v) solution of methanol and 100 mM ammonium bicarbonate. The buffer was exchanged, and the samples were destained for another 15 minutes. This was repeated for another 3 cycles. The gel pieces were dehydrated by washing with acetonitrile and further dried using a SpeedVac for 20 minutes. Protein reduction and alkylation were performed by reswelling the gel in 50 mM ammonium bicarbonate with 20 mM DTT for 1 hour at 57°C. This solution was replaced by 50 mM ammonium bicarbonate containing 50 mM iodoacetamide for 45 minutes at room temperature in the dark. The gel pieces were dehydrated using acetonitrile as described above. The dehydrated gel pieces were incubated with 250 ng of sequencing-grade modified trypsin (Promega) in enough 100 mM ammonium bicarbonate to cover the gel pieces. After overnight digestion with gentle agitation at room temperature, a slurry of R2 50-μm Poros beads (Applied Biosystems) in 5% formic acid and 0.2% trifluoroacetic acid (TFA) was added to each sample at a volume equal to that of the ammonium bicarbonate added for digestion as previously described [61]. The samples were allowed to shake at 4° C for 2 hours. The beads were loaded onto C18 ziptips (Millipore), equilibrated with 0.1% TFA by centrifuging in a microcentrifuge for 30 seconds at 6000 RPM. The beads were washed with 0.5% acetic acid. Peptides were eluted with 40% acetonitrile in 0.5% acetic acid followed by 80% acetonitrile in 0.5% acetic acid. The organic solvent was removed using a SpeedVac concentrator, and the sample was reconstituted in 0.5% acetic acid.

### Mass spectrometry analysis

An aliquot of the sample was loaded onto an Acclaim PepMap trap column (2 cm x 75 μm) in line with an EASY-Spray analytical column (50 cm x 75 μm ID PepMap C18, 2 μm bead size) using the auto sampler of an EASY-nLC 1000 HPLC (Thermo Fisher Scientific) with solvent A consisting of 2% acetonitrile in 0.5% acetic acid and solvent B consisting of 80% acetonitrile in 0.5% acetic acid. The peptides were eluted into an Orbitrap Q Exactive Mass Spectrometer (Thermo Fisher Scientific) using the following gradient: 5–35% B in 60 minutes, 35–45% B in 10 minutes, followed by 45–100% B in 10 minutes. High-resolution mass spectra were recorded with a resolution of 70,000, an AGC target of 1e6, with a maximum ion time of 120 ms, and a scan range from 400 to 1500 m/z. Following each full scan, twenty data-dependent MS/MS spectra were acquired. The MS/MS spectra were collected with a resolution of 17,500, an AGC target of 5e4, maximum ion time of 120 ms, one microscan, 2 m/z isolation window, fixed first mass of 150 m/z, dynamic exclusion of 30 sec, and normalized collision energy (NCE) of 27.

### Mass spectrometry data processing

All acquired MS2 spectra were searched against a UniProt *Vibrio cholerae* and a VpsO only database using Byonic [62], and phosphorylated VpsO spectra were manually verified. The search parameters were as follows: precursor mass tolerance ±10 ppm, fragment mass tolerance ± 0.02 Da, digestion parameters allowing 2 trypsin missed cleavages, fixed modification of carbamidomethyl on cysteine, variable modification of oxidation on methionine, variable modification of deamidation on glutamine and asparagine, and variable modification of phosphorylation on serine, tyrosine, and threonine. The results were filtered to only include peptides identified with a Byonic Score above 300. Phosphorylated peptides were manually verified.

VpsO-Myc/His was digested with two different proteases, trypsin (T) and GluC (C) endoproteinase, to maximize sequence coverage. Despite the digestion with two proteases certain tyrosines were not covered: Y49 (T peptide not detected, C peptide not enough sequence coverage), Y394 (T peptide was too small to fly, C peptide not enough sequence coverage), Y607 (T peptide was too large to fly, most likely no C cleavage due peptide charge distance), Y646 (T peptide was too small to fly, C peptide was too large to fly).

### Analysis of biofilm architecture

For single-cell resolution microscopy of biofilms, the GFP-expression plasmid pNUT542 was introduced into the relevant strains, which conferred gentamycin resistance. The strains were grown in M9 minimal medium, supplemented with 0.5% (w/v) glucose and 30 μg/ml gentamycin, to mid-exponential growth phase, before introducing them into microfluidic channels of dimensions 500 μm x 100 μm x 7000 μm (width x height x length). After the cultures were introduced into the channels, the channels were incubated at 24° C for 1 hour without flow to allow cells to attach to the bottom glass surface of the channels. The flow was then set to 0.1 μl/min, corresponding to an average flow speed of 33 μm/s in the channels, for approximately 21 hours before images were acquired. Images were acquired with a silicon oil immersion objective (Olympus 100x, numerical aperture of 1.35) using a Yokogawa spinning disk confocal scanner and laser excitation at 488 nm. Images were acquired at spatial resolution of 63 nm in the *xy* plane and 400 nm along the *z* direction. Single-cell resolution microscopy was performed on biofilm microcolonies with three biological replicates per strain. To reduce phototoxicity and photobleaching, an adaptive microscope control algorithm was used during image acquisition as described previously [63].

To detect all single cells and measure architectural properties of the biofilms grown in flow chambers, images were analysed using Matlab as described in [63,64]. Briefly, cells were automatically identified using edge detection algorithms, and then a watershed algorithm. Cellular orientations were obtained by mapping an ellipsoid onto each cell using principal component analysis. For all cells, the vector of the major cell axis was used to determine the value of the local nematic order parameter defined as *S* = <3/2 (**n**_*i*_.**n**_*j*_)^2^ −1/2>, where **n**_*i*_ is the orientation vector of a particular cell and **n**_*j*_ are the orientation vectors of cells in the local vicinity, within a local vicinity defined by a sphere of radius 3 μm around each cell. A high nematic order parameter *S* indicates that the cells are locally highly aligned, whereas a low value of *S* indicates a disordered cellular arrangement. We also determined the alignment of each cell in the vertical direction by computing the angle of each cell with the vertical *z*-axis. The average centre-to-centre distance of each cell to its nearest neighbor was calculated for each cell based on cell-centroid coordinates.

### Biofilm competition analysis

For competition assays rugose/mutant::Tn7-GFP strains were combined at a dilution of 1:400 individually, with a 1:400 dilution of rugose::Tn7-RFP cells in 1 mL of 2% LB media, and 200 μL of the strain mixtures were pipetted into channels of an μ-Slide VI 0.4 uncoated plastic bottom slide (Ibidi). Cells were allowed to attach for 1 hour at room temperature, and flow of 2% LB media was established at a rate of ~8 mL per channel per hour. Biofilms were allowed to form at room temperature. Images of the developing biomass were obtained on a Zeiss LSM 880 confocal microscope at 1, 3, 6, and 24 hours post establishment of flow (post-flow), at 10x magnification for biomass analysis and 40x magnification for image generation. Images were processed with Imaris (Oxford Instruments), and biomass quantification was performed using Comstat2 [65]. Biomass levels for both the rugose::RFP and mutant::GFP channels were determined using Comstat2. The percentage of the mutant::GFP biomass relative to total biomass was determined at each time point and was plotted as the mean of two biological replicates imaged at 10x magnification (with three technical replicates per biological replicate and time point) with error bars indicating the standard error of the mean.

### Scanning electron microscopy analysis of cell shape

20 ml of LB agar was added per plate, and the plates were dried at room temperature for one day before use. To grow the cells, 3 μl of well-vortexed overnight culture was spotted on the plate and grown for ~24 hours at 25°C. The agar was cut around the spots, so the agar cubes containing the spots could be fixed in 2.5% glutaraldehyde in PBS (pH 7.4) for 1 hour at room temperature. Samples were dehydrated with 30%, 50%, 70%, and 90% ethanol dilutions and then stored overnight in 100% ethanol (Sigma Aldrich) at room temperature. The samples were critically point dried (model: Balzers Union 342/11 120B), sputtered with ~20 nm of gold (model: Technics Hummer VI), and were visualized with a FEI Quanta 3D Dualbeam SEM operating at 5 kV and 6.7 pA at the SEM facility at University of California Santa Cruz.

### Phase contrast microscopy analysis of cell shape

For analysis of cell shape, overnight stationary phase rugose and Δ*vpsO* cells were analyzed on a Zeiss Axiovert 200 phase contrast microscope outfitted with a CoolSNAP HQ2 monochrome CCD camera (Photometrics) at 63x magnification. Cell length and mean cell width were then calculated and plotted using MicrobeJ software for ImageJ [66].

### Live-dead staining

Strains of rugose, Δ*vps-I*Δ*vps-II*, and Δ*vpsO* were grown in independent flow cell chambers. After 5 hours, the flow cell chamber was disconnected from flow. Growth media was pipetted from the chamber, and each chamber was washed 1x with defined artificial seawater (DASW) media. After washing, chambers were stained for live/dead cells with SYTO 9 (live cells) and propidium iodide (dead cells) and suspended in DASW for 15 minutes at room temperature (BacLight Bacterial Viability Kit, Invitrogen). Chambers were then analyzed on a Zeiss LSCM 880 microscope at 40x magnification. The microscope was equipped with a TMPT detector for simultaneous acquisition of a pseudo-bright-field image (TPMT). Images were processed and channels merged using Image J.

### Vancomycin sensitivity analysis

As previously reported 2 μl aliquots of an overnight culture grown at 30°C were spotted on 20 ml LB and LB/ vancomycin (200 μg/ml) agar plates, grown for 24 hours and then imaged [49].

## Supporting information

S1 Fig Data supporting the characterization of VpsO and VpsU structure and activity. **(A)** Incorporation of the radiolabeled phosphate from [γ-^32^P]-ATP into VpsO kinase domain constructs VpsO-473 (residues 473–737) and VpsO-503 (503–737); n≥2. Phosphorimage of the SDS-PAGE gel with analyzed proteins is shown. **(B)** Depicts the deconvoluted mass spectra for VpsO-503, VpsO-503^K551A^, and VpsO-503^E519A, R522A, R525A^. Both the VpsO-503 and VpsO-503^E519A, R522A, R525A^ mutant are heterogeneously phosphorylated (series of peaks separated by 80 Da), while the K551A mutant shows a single peak corresponding to the unphosphorylated molecular weight. **(C)** Dimerization of VpsO-503^E519A, R522A, R525A^ in the crystal structure. Two copies of the VpsO kinase domain mutant protein are present in the asymmetric unit. The dimer structure is consistent with the apparent size of the mutant protein in solution as assayed by SEC-MALS (Fig 6). The dimerization interface is formed by the α1 helix in the kinase domain. This helix contains the E519A, R522A, R525A mutations that break oligomerization, so it is not clear whether the wild-type protein forms the same dimer interface *in vivo*. **(D)** Phos-Tag SDS-PAGE analysis of WT VpsO-503 in the presence and absence of WT VpsU and the catalytically inactive VpsU^C12S^ mutant; n≥2. P refers to the tyrosine phosphorylated state and U refers to the unphosphorylated state. Proteins are stained with Coomassie dye. The band marked with an asterisk is a contaminant in the VpsU^C12S^ preparation. The VpsO-503 in this experiment is initially heterogeneously phosphorylated following recombinant expression and purification.
(TIFF)

S2 Fig Analysis of *in vivo* VpsO phosphorylation sites. **(A)** Diagram of VpsO domain organization and sites of phosphorylation as determined by mass spectrometry analysis of VpsO-Myc/His expressed in *V*. *cholerae*. **(B)** Mass spectrum of a periplasmic and **(C)** a cytoplasmic tyrosine-phosphorylated peptide, respectively.
(TIFF)

S3 Fig *In vivo* tyrosine phosphorylation analysis of strains harboring Y to F substitutions in the VpsO C-terminal tail. **(A)** Diagram of VpsO highlighting domain organization and the positions of the potential phosphorylation sites. **(B, C)** Western blot analysis for VpsO abundance and tyrosine phosphorylation; n≥3.
(TIFF)

S4 Fig *In vivo* tyrosine phosphorylation analysis of strains harboring Y to F substitutions in the VpsO periplasmic region. **(A)** Diagram of VpsO showing the positions of the phosphorylation sites. **(B)** Western blot analysis for VpsO abundance and tyrosine phosphorylation; n≥3.
(TIFF)

S5 Fig VpsO is the only tyrosine phosphorylated Vps protein encoded by the *vps* clusters. pBAD-*vpsU-Q*-Myc/His constructs were screened for protein expression and tyrosine phosphorylation in their respective deletion backgrounds.
(TIFF)

S6 Fig Growth of the Δ*vpsO* strain under flow induces a rounded cell phenotype. **(A)** Representative scanning electron microscopy images of rugose, Δ*vps-I*Δ*vps-II*, and Δ*vpsO* cells grown as spot-colonies. **(B)** Representative phase contrast images of stationary phase rugose and Δ*vpsO* cells taken at 63x magnification. Scale bars = 2 μm. Graph is the MicobeJ quantification of the cell length plotted against the mean cell width for each strain. A total of 3883 rugose (cyan) and 4556 Δ*vpsO* (yellow) cells were analyzed from three individual images of each strain obtained from one biological replicate. **(C)** Live/dead staining of rugose, Δ*vps-I*Δ*vps-II*, and Δ*vpsO* cells grown under flow. Unlabeled strains were grown in independent flow cell chambers for 5-hours. Representative images of live (green) and dead (red) cells and a pseudo-bright-field image are shown. Scale bars = 4 μm.
(TIFF)

S7 Fig VpsO-503 does not undergo tyrosine trans autophosphorylation *in vitro*. **(A)** [γ-^32^P]-ATP kinase assay as in Fig 5E. Where indicated, VpsO-503 was incubated with the catalytically inactive GST-VpsO-503^K551A^. The mutant was purified as a GST fusion such that it has different mobility on the SDS-PAGE gel. The lack of a second band at a slower migration indicates that we did not observe *in trans* phosphorylation on the C-terminal tail of the GST-fusion VpsO-503^K551A^ (catalytically inactive) mutant by the VpsO-503 WT enzyme. **(B)** [γ-^32^P]-ATP kinase assay as in Fig 5E. 50 μM VpsO-503 WT or VpsO-503^E519A, R522A, R525A^ was incubated in the absence or presence of increasing amounts of catalytically inactive VpsO-503^K551A^ (from 7 to 55 μM in 2-fold increments) for 1 hour and then reacted with ATP for 30 minutes. The observation that catalytic activity does not decrease with addition of the catalytically inactive enzyme suggests that the observed phosphorylation does not occur *in trans* within an oligomer.
(TIFF)

S1 Video Rounded live Δ*vpsO* cells detach from the surface. Composite overlay of images of live (green) and dead (red) Δ*vpsO* cells obtained every 3 seconds over the course of 60 seconds, played back at 3 frames-per-second (fps). Scale bar = 20μm.
(AVI)

S1 Table X-ray crystallography data collection and refinement statistics. Values in parentheses are for the highest resolution shell.
(PDF)

S2 Table VpsO tyrosine phosphorylated peptides. (PDF)

S3 Table Vps genes and their expected protein sizes. (PDF)

S4 Table Quantification of surface colonization, micro-colony size and biomass during competition biofilm formation experiment. (PDF)

S5 Table Strains and plasmids used in this study. (PDF)

S1 Data Primary data used for analysis. (XLSX)
