# Supplementary figures and images for: A tyrosine phosphoregulatory system controls exopolysaccharide biosynthesis and biofilm formation in Vibrio cholerae
Source: PLoS Pathog. 2020 Aug 25;16(8):e1008745. doi: 10.1371/journal.ppat.1008745 (PMC7485978; doi:10.1371/journal.ppat.1008745)

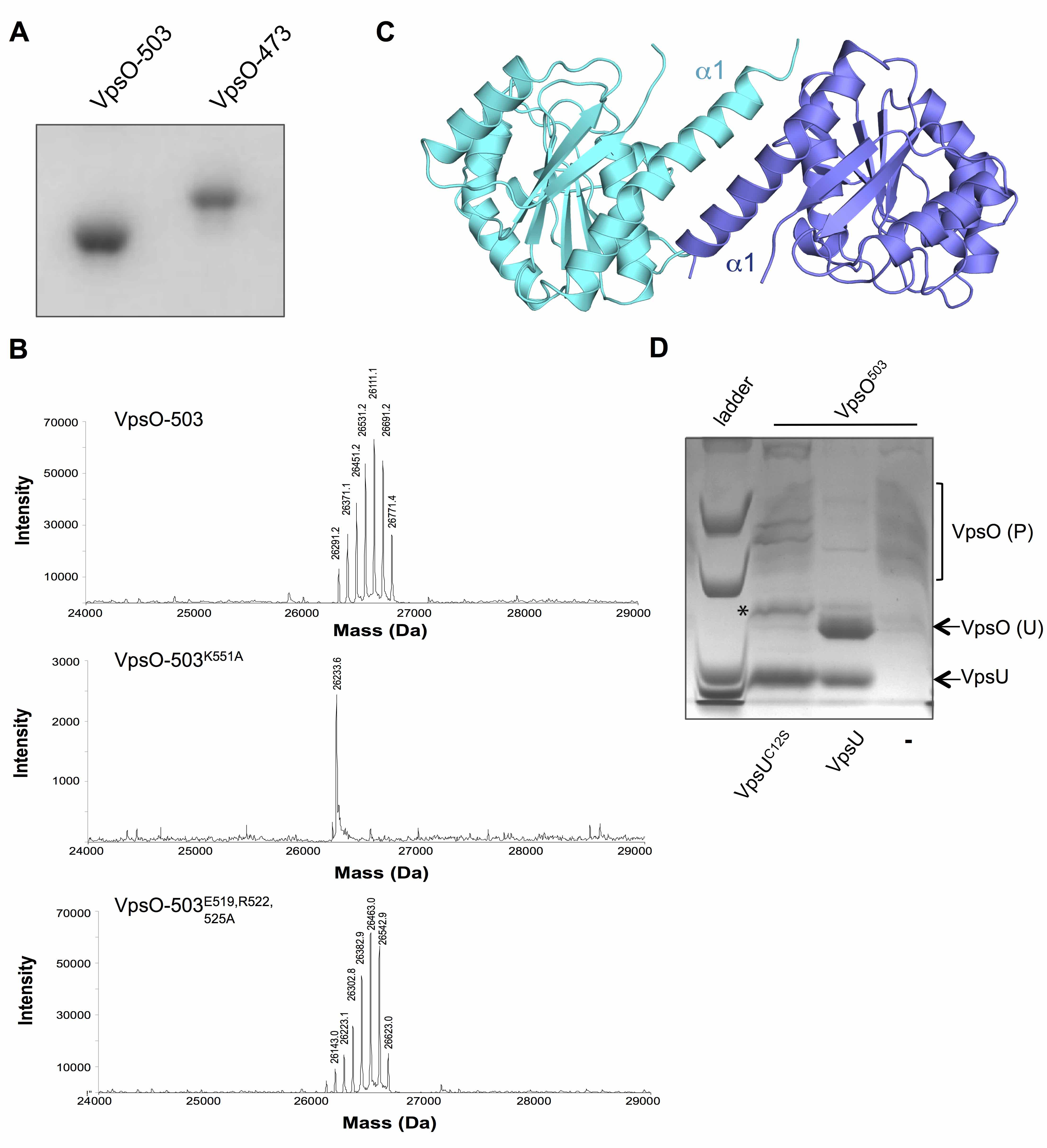

Supplement: S1 Fig — (A) Incorporation of the radiolabeled phosphate from [γ-32P]-ATP into VpsO kinase domain constructs VpsO-473 (residues 473–737) and VpsO-503 (503–737); n≥2. Phosphorimage of the SDS-PAGE gel with analyzed proteins is shown. (B) Depicts the deconvoluted mass spectra for VpsO-503, VpsO-503K551A, and VpsO-503E519A, R522A, R525A. Both the VpsO-503 and VpsO-503E519A, R522A, R525A mutant are heterogeneously phosphorylated (series of peaks separated by 80 Da), while the K551A mutant shows a single peak corresponding to the unphosphorylated molecular weight. (C) Dimerization of VpsO-503E519A, R522A, R525A in the crystal structure. Two copies of the VpsO kinase domain mutant protein are present in the asymmetric unit. The dimer structure is consistent with the apparent size of the mutant protein in solution as assayed by SEC-MALS (Fig 6). The dimerization interface is formed by the α1 helix in the kinase domain. This helix contains the E519A, R522A, R525A mutations that break oligomerization, so it is not clear whether the wild-type protein forms the same dimer interface in vivo. (D) Phos-Tag SDS-PAGE analysis of WT VpsO-503 in the presence and absence of WT VpsU and the catalytically inactive VpsUC12S mutant; n≥2. P refers to the tyrosine phosphorylated state and U refers to the unphosphorylated state. Proteins are stained with Coomassie dye. The band marked with an asterisk is a contaminant in the VpsUC12S preparation. The VpsO-503 in this experiment is initially heterogeneously phosphorylated following recombinant expression and purification. (TIFF) [file ppat.1008745.s001.tiff]

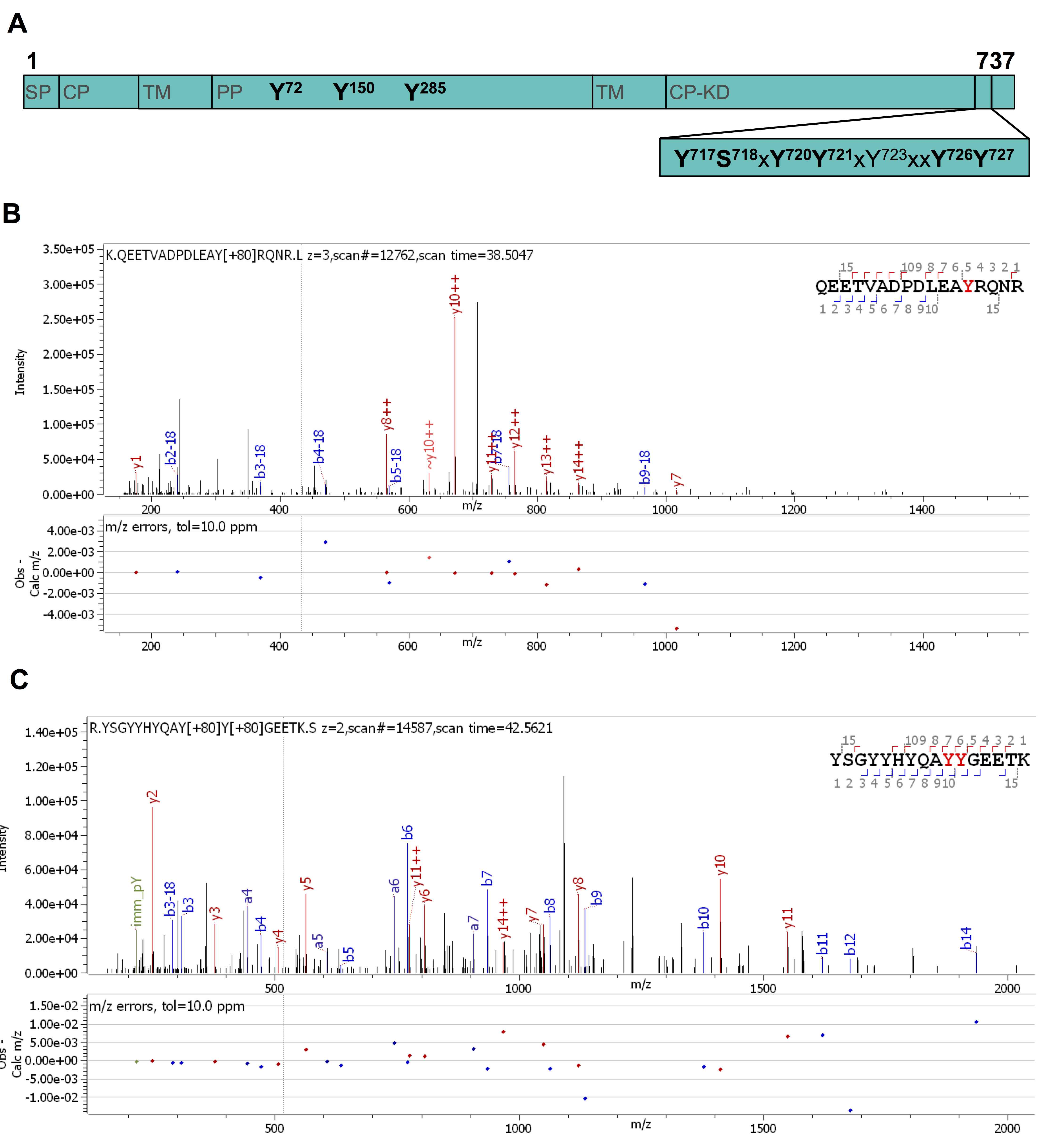

Supplement: S2 Fig — (A) Diagram of VpsO domain organization and sites of phosphorylation as determined by mass spectrometry analysis of VpsO-Myc/His expressed in V. cholerae. (B) Mass spectrum of a periplasmic and (C) a cytoplasmic tyrosine-phosphorylated peptide, respectively. (TIFF) [file ppat.1008745.s002.tiff]

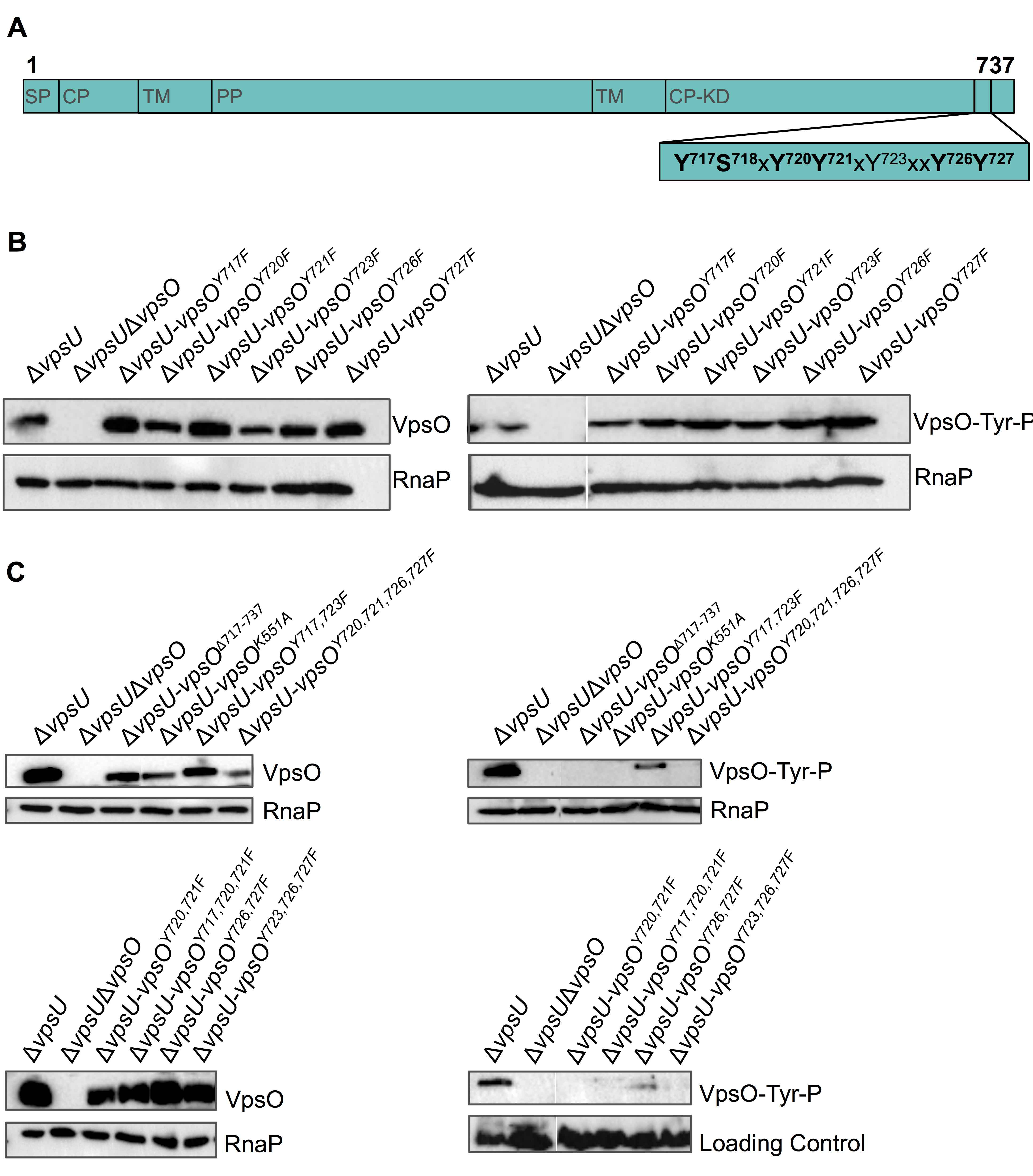

Supplement: S3 Fig — (A) Diagram of VpsO highlighting domain organization and the positions of the potential phosphorylation sites. (B, C) Western blot analysis for VpsO abundance and tyrosine phosphorylation; n≥3. (TIFF) [file ppat.1008745.s003.tiff]

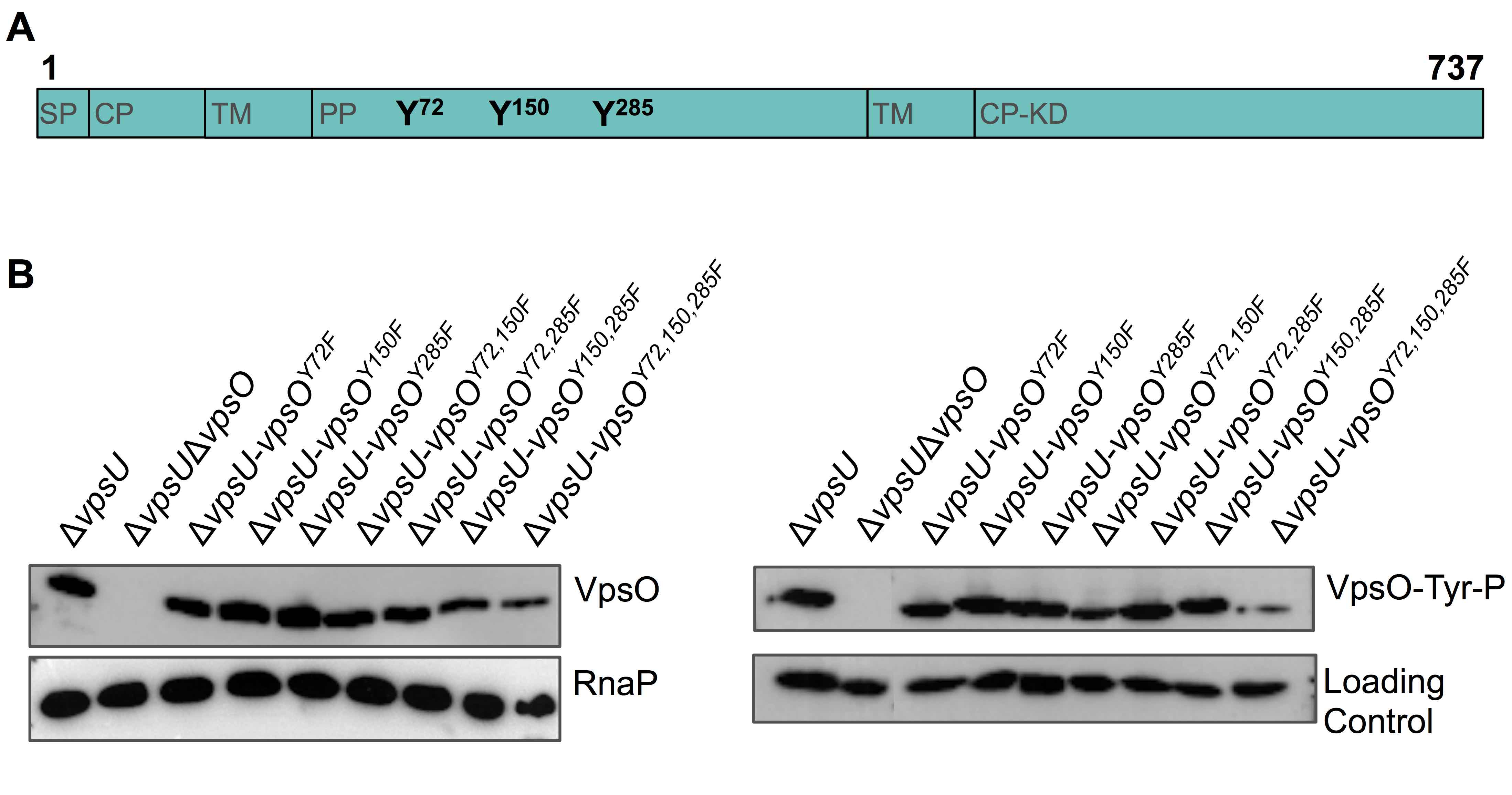

Supplement: S4 Fig — (A) Diagram of VpsO showing the positions of the phosphorylation sites. (B) Western blot analysis for VpsO abundance and tyrosine phosphorylation; n≥3. (TIFF) [file ppat.1008745.s004.tiff]

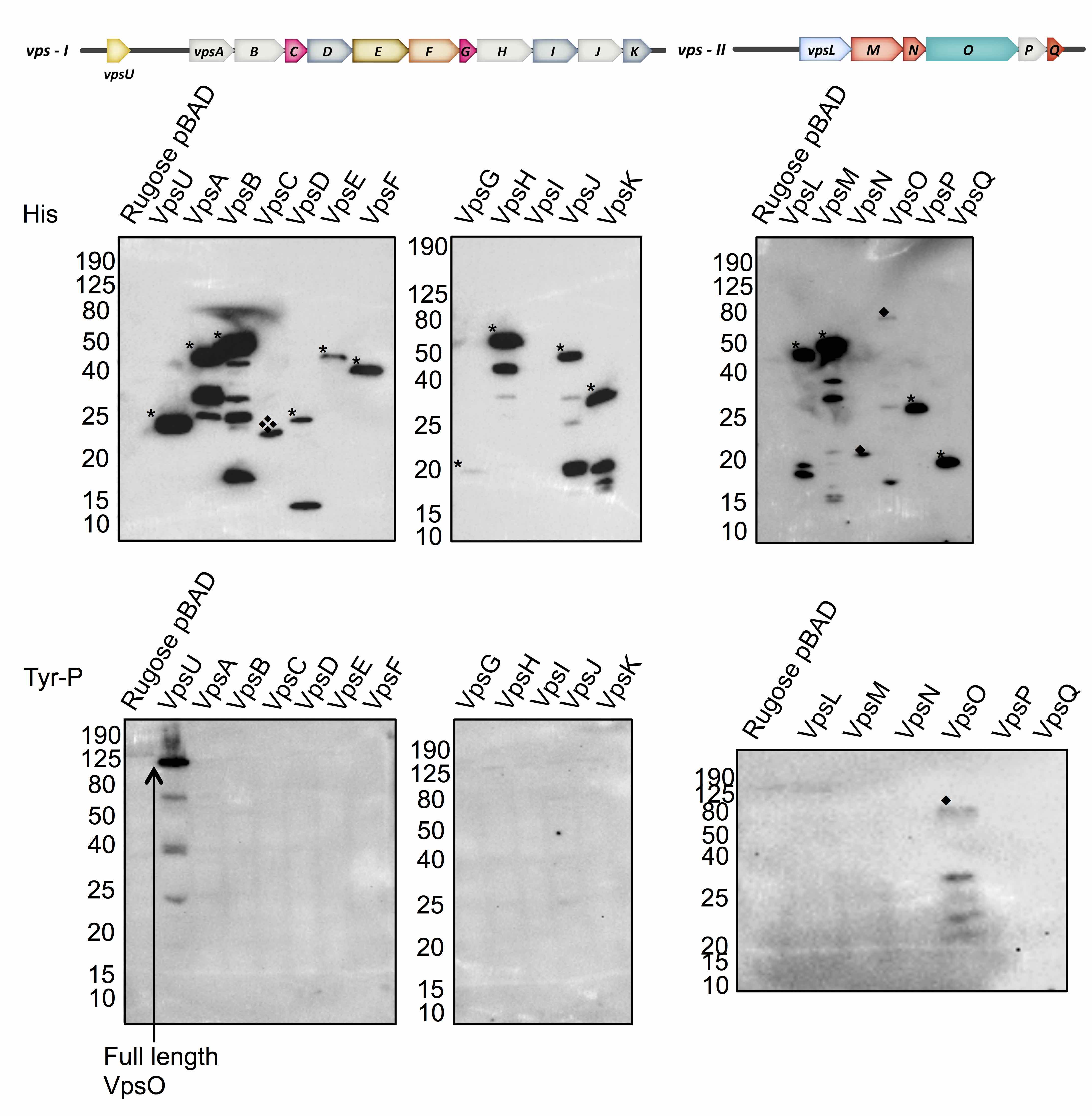

Supplement: S5 Fig — pBAD-vpsU-Q-Myc/His constructs were screened for protein expression and tyrosine phosphorylation in their respective deletion backgrounds. (TIFF) [file ppat.1008745.s005.tiff]

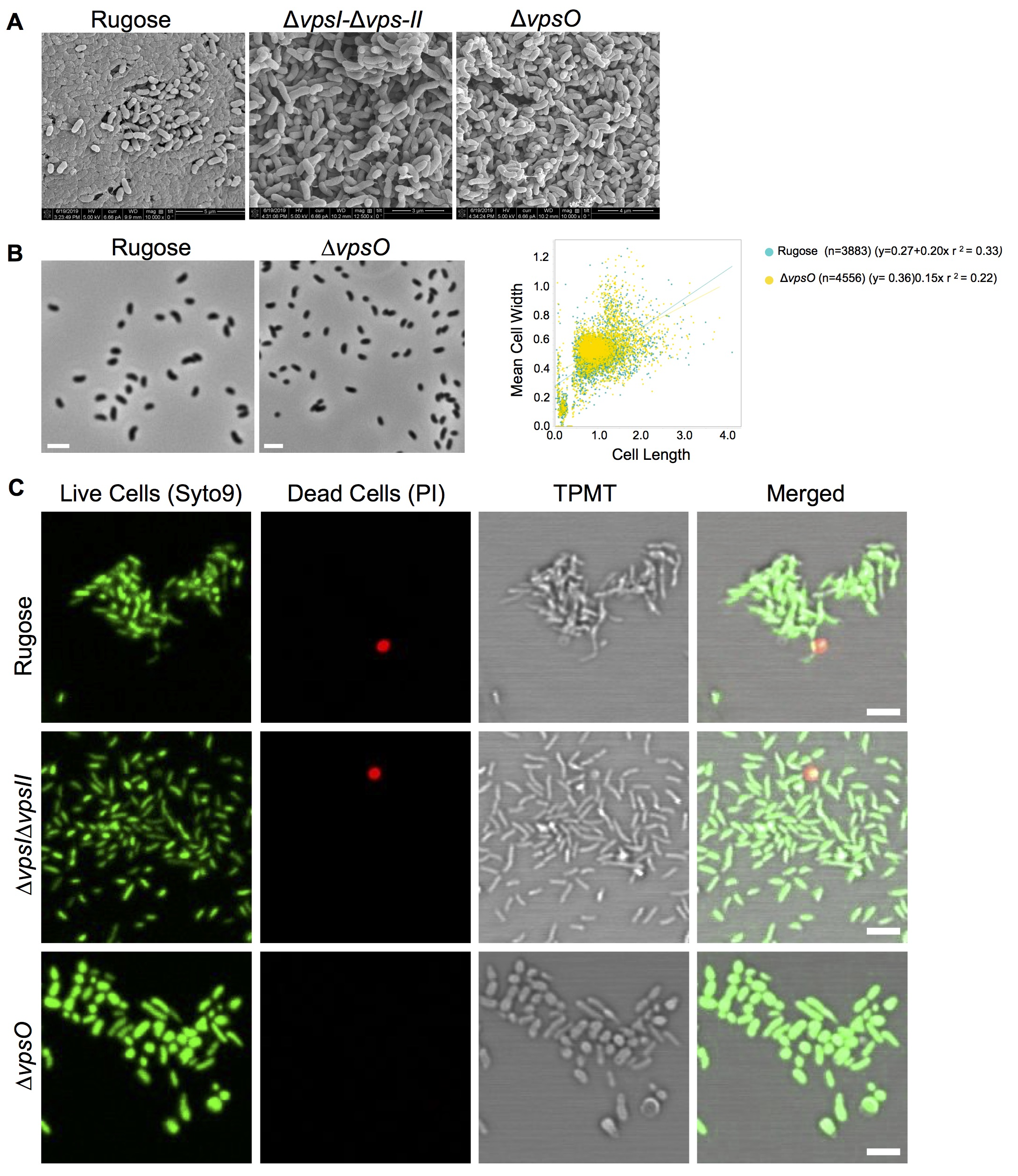

Supplement: S6 Fig — (A) Representative scanning electron microscopy images of rugose, Δvps-IΔvps-II, and ΔvpsO cells grown as spot-colonies. (B) Representative phase contrast images of stationary phase rugose and ΔvpsO cells taken at 63x magnification. Scale bars = 2 μm. Graph is the MicobeJ quantification of the cell length plotted against the mean cell width for each strain. A total of 3883 rugose (cyan) and 4556 ΔvpsO (yellow) cells were analyzed from three individual images of each strain obtained from one biological replicate. (C) Live/dead staining of rugose, Δvps-IΔvps-II, and ΔvpsO cells grown under flow. Unlabeled strains were grown in independent flow cell chambers for 5-hours. Representative images of live (green) and dead (red) cells and a pseudo-bright-field image are shown. Scale bars = 4 μm. (TIFF) [file ppat.1008745.s006.tiff]

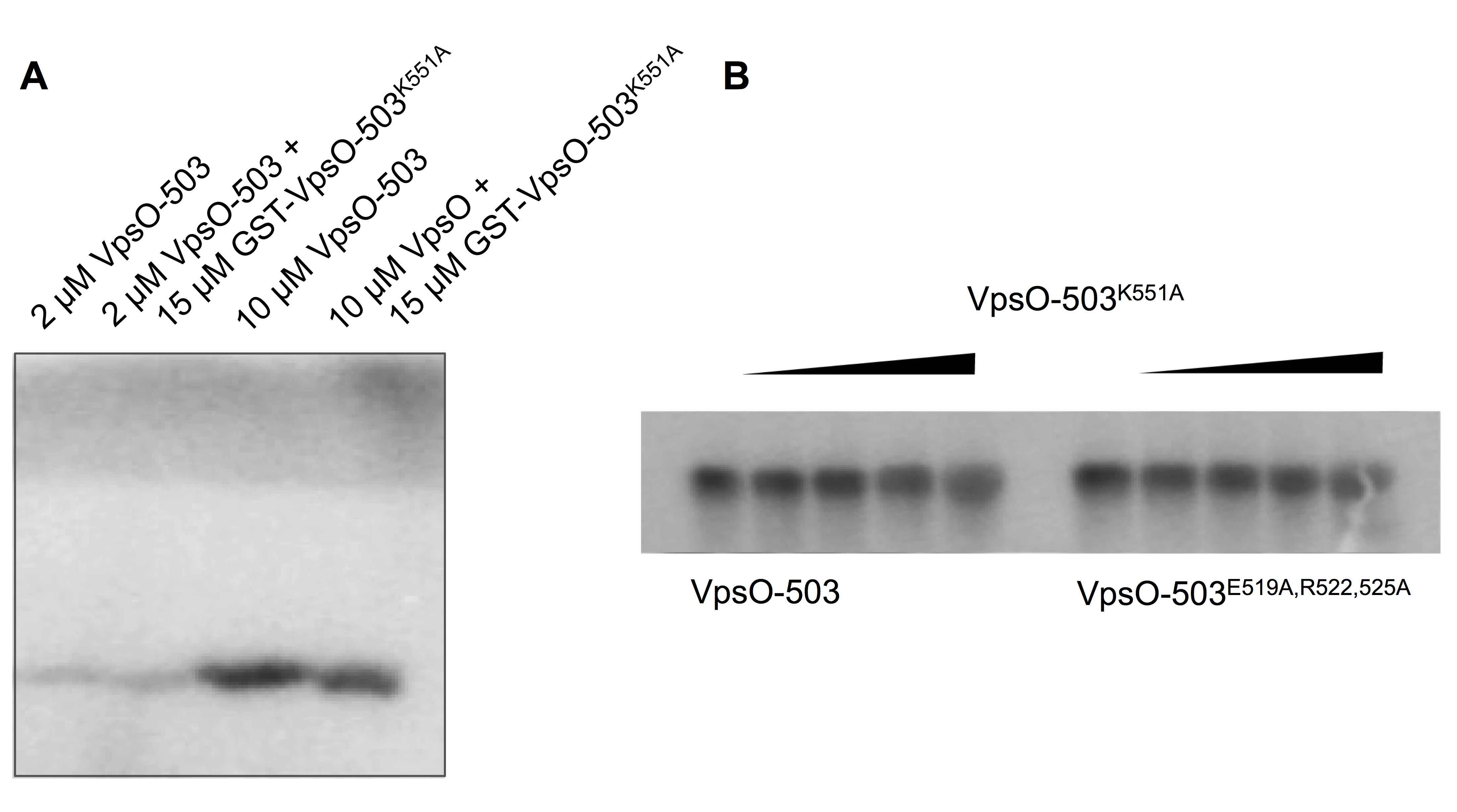

Supplement: S7 Fig — (A) [γ-32P]-ATP kinase assay as in Fig 5E. Where indicated, VpsO-503 was incubated with the catalytically inactive GST-VpsO-503K551A. The mutant was purified as a GST fusion such that it has different mobility on the SDS-PAGE gel. The lack of a second band at a slower migration indicates that we did not observe in trans phosphorylation on the C-terminal tail of the GST-fusion VpsO-503K551A (catalytically inactive) mutant by the VpsO-503 WT enzyme. (B) [γ-32P]-ATP kinase assay as in Fig 5E. 50 μM VpsO-503 WT or VpsO-503E519A, R522A, R525A was incubated in the absence or presence of increasing amounts of catalytically inactive VpsO-503K551A (from 7 to 55 μM in 2-fold increments) for 1 hour and then reacted with ATP for 30 minutes. The observation that catalytic activity does not decrease with addition of the catalytically inactive enzyme suggests that the observed phosphorylation does not occur in trans within an oligomer. (TIFF) [file ppat.1008745.s007.tiff]
