# Supplementary material for: A tyrosine phosphoregulatory system controls exopolysaccharide biosynthesis and biofilm formation in Vibrio cholerae
Source: PLoS Pathog. 2020 Aug 25;16(8):e1008745. doi: 10.1371/journal.ppat.1008745 (PMC7485978; doi:10.1371/journal.ppat.1008745)
Supplement: S1 Table — Values in parentheses are for the highest resolution shell. (PDF) [file ppat.1008745.s009.pdf]

S1 Table. X-ray crystallography data collection and refinement statistics. Values in parentheses are for the highest resolution shell.

	**VpsO (503-737, E519, R522, R525)**	**VpsU**
Data collection		
Space group	P 64	I 41
Resolution (Å)	44.39-2.87 (3.03-2.87)	63.81-2.20 (2.27-2.20)
Cell dimensions		
a, b, c (Å)	171.43, 171.43, 44.39	80.35, 80.35, 105.06
α, β, γ (°)	90, 90, 120	90, 90, 90
R_{merge} (%)	9.6 (66.0)	6.8 (72.4)
Total reflections	80309(11088)	142908 (5209)
Unique reflections	17506 (2518)	16904 (1449)
I/σ	9.2 (2.5)	18.2 (2.8)
$CC_{1/2}$	0.99 (0.73)	0.99(0.56)
Completeness (%)	100 (100)	99.9(98.8)
Redundancy	4.6 (4.4)	8.5 (3.6)
Refinement		
R_{work}/R_{free}	17.5/22.7	18.8/25.7
No. of atoms	3179	2420
Protein	3179	2373
Water	-	35
RMS deviation		
Bond lengths (Å)	0.01	0.008
Bond angles (°)	1.22	1.39
Ramachandran		
Favored/Outliers (%)	100.0/0.0	100/0.0
B-Factor (Å^2)	74	64
PDB accession code	6U1Q	6U1P
