# Supplementary material for: A tyrosine phosphoregulatory system controls exopolysaccharide biosynthesis and biofilm formation in Vibrio cholerae
Source: PLoS Pathog. 2020 Aug 25;16(8):e1008745. doi: 10.1371/journal.ppat.1008745 (PMC7485978; doi:10.1371/journal.ppat.1008745)
Supplement: S2 Table — (PDF) [file ppat.1008745.s010.pdf]

S2 Table. VpsO tyrosine phosphorylated peptides.

Peptide Sequence	Position in the Protein	Area Under The Curve
AVSIEEVYGLDTK		7.22E+10
AVSIEEVyGLDTK	71	7.46E+07
**Total Area**		7.23E+10
**Relative Phosphorylation (%)**		**0.10**
QEETVADPDLEAYRQNR		3.62E+08
QEETVADPDLEAyRQNR	149	9.39E+06
**Total Area**		3.71E+08
**Relative Phosphorylation (%)**		**2.53**
VAAETLYQVANSYSK		5.10E+10
VAAETLYQVANSySK	284	1.33E+07
**Total Area**		5.10E+10
**Relative Phosphorylation (%)**		**0.03**
YSGYYHYQAYYGEETK		4.99E+10
*YsGYYHYQAyYGEETK	717,725	2.78E+08
ySGYYHYQAYYGEETK	716	2.21E+09
YSGyYHYQAYYGEETK	719	9.01E+09
YSGYyHYQAYYGEETK	720	8.95E+09
YSGYYHyQAYYGEETK	722	6.67E+09
YSGYYHYQAyYGEETK	725	8.93E+09
YSGYYHYQAYyGEETK	726	8.88E+09
ySGYYHYQAYyGEETK	716,726	6.62E+08
YSGyYHYQAyYGEETK	719,725	6.62E+08
YSGyYHYQAYyGEETK	719,726	9.26E+08
YSGYYHYQAyyGEETK	725,726	9.26E+08
**Total Area**		9.80E+10
**Relative Phosphorylation (%)**		**49.11**

* This peptide containing 16 residues has 8 residues that can be phosphorylated. We therefore performed a targeted analysis to confirm the site specific phosphorylation and used the rough areas on slide 9 to get an approximate area for a specific modified residue. However, this is difficult to determine as these are mixed spectra due to co-elution of the different phosphor-isoforms.
