# Supplementary material for: A tyrosine phosphoregulatory system controls exopolysaccharide biosynthesis and biofilm formation in Vibrio cholerae
Source: PLoS Pathog. 2020 Aug 25;16(8):e1008745. doi: 10.1371/journal.ppat.1008745 (PMC7485978; doi:10.1371/journal.ppat.1008745)
Supplement: S3 Table — (PDF) [file ppat.1008745.s011.pdf]

S3 Table. Vps genes and their expected protein sizes

Gene Number	Name	Expected Size (kDa)	Actual Size	Difference between N1961 & A1552
VC0916	*vpsU*	23.5		
VC0917	*vpsA*	43.5		
VC0918	*vpsB*	47		
VC0919	*vpsC*	11.3	~22.5	Insertion in A1552
VC0920	*vpsD*	46.3		
VC0921	*vpsE*	54.6		
VC0922	*vpsF*	47.6		
VC0923	*vpsG*	17.9		
VC0924	*vpsH*	53.2		
VC0925	*vpsI*	43		
VC0926	*vpsJ*	47.2		
VC0927	*vpsK*	31.1		
VC0934	*vpsL*	55.6		
VC0935	*vpsM*	47.5		
VC0936	*vpsN*	22.5		
VC0937	*vpsO*	85.5		
VC0938	*vpsQ*	29.2		
VC0939	*vpsP*	18.5		
