# Supplementary material for: A tyrosine phosphoregulatory system controls exopolysaccharide biosynthesis and biofilm formation in Vibrio cholerae
Source: PLoS Pathog. 2020 Aug 25;16(8):e1008745. doi: 10.1371/journal.ppat.1008745 (PMC7485978; doi:10.1371/journal.ppat.1008745)
Supplement: S4 Table — (PDF) [file ppat.1008745.s012.pdf]

S4 Table. Quantification of surface colonization, micro-colony size and biomass during competition biofilm formation experiment..

Strain / Treatment	Biomass ($\mu m^3/\mu m^2$)		Micro-colonies at Substratum			
			Total Number		Avg. Volume (μm^2)	
	Competitor	Rugose	Competitor	Rugose	Competitor	Rugose
1 Hour						
**Rugose vs. Rugose**	0.0095 (0.0011)	0.0079 (0.0006)	5	3	109.78 (9.02)	183.19 (147.46)
**Δ*vpsI*Δ*vpsII* vs. Rugose**	0.0205 (0.0019)	0.0125 (0.0025)	0	12	n/a	112.94 (0.16)
**Δ*vpsO* vs. Rugose**	0.0104 (0.0111)	0.0092 (0.0076)	0	4	n/a	160.62 (43.87)
***vpsO*^{Y720,721,726,727F} vs. Rugose**	0.0268 (0.0182)	0.0185 (0.0148)	0	9	n/a	133.73 (23.88)
***vpsO*^{K551A} vs. Rugose**	0.0138 (0.0089)	0.0137 (0.0099)	0	2	n/a	113.40 (16.09)
**Δ*vpsU* vs. Rugose**	0.0174 (0.0012)	0.0106 (0.0009)	1	10	115.12 (n/a)	154.36 (31.28)
6 Hours						
**Rugose vs. Rugose**	2.561 (0.448)	1.473 (0.059)	260	41	164.14 (17.91)	336.16 (157.62)
**Δ*vpsI*Δ*vpsII* vs. Rugose**	7.245 (1.456)	4.559 (0.549)	473	30	269.00 (62.15)	299.18 (147.77)
**Δ*vpsO* vs. Rugose**	1.575 (0.288)	1.577 (0.847)	111	38	109.49 (12.65)	348.24 (89.76)
***vpsO*^{Y720,721,726,727F} vs. Rugose**	5.720 (3.456)	1.511 (0.523)	275	34	231.39 (42.70)	309.07 (68.25)
***vpsO*^{K551A} vs. Rugose**	7.461 (6.174)	3.646 (3.201)	275	123	410.62 (290.39)	173.76 (22.10)
**Δ*vpsU* vs. Rugose**	4.559 (0.549)	1.763 (0.339)	492	35	211.02 (18.17)	299.68 (61.34)
24 Hours						
**Rugose vs. Rugose**	20.61 (2.98)	12.98 (1.38)	-	-	-	-
**Δ*vpsI*Δ*vpsII* vs. Rugose**	8.52 (2.44) *	20.51 (1.84)	-	-	-	-
**Δ*vpsO* vs. Rugose**	8.87 (1.58) *	22.39 (2.77)	-	-	-	-
***vpsO*^{Y720,721,726,727F} vs. Rugose**	11.94 (3.01)	16.21 (0.007)	-	-	-	-
***vpsO*^{K551A} vs. Rugose**	27.96 (9.78) **	9.57 (0.83)	-	-	-	-
**Δ*vpsU* vs. Rugose**	21.21 (2.34)	12.73 (1.20)	-	-	-	-

Figure 10 – table supplement 1. Comstat2 quantification values for flow cell biofilm competition model.

Values were determined from two biological replicates, each with three 20x images of each competition at each time point, using Comstat2 software for ImageJ. Statistics were performed using GraphPad Prism version 8.1.1. Statistical significance determined via Student's t-Test comparing the competitor with rugose for each strain competition (within an individual time-point) using the Holm-Sidak method, with alpha = 0.05. *p ≤ 0.05, **p ≤ 0.001, ***p ≤ 0.0001, ****p ≤ 0.00001.
