# Supplementary material for: A tyrosine phosphoregulatory system controls exopolysaccharide biosynthesis and biofilm formation in Vibrio cholerae
Source: PLoS Pathog. 2020 Aug 25;16(8):e1008745. doi: 10.1371/journal.ppat.1008745 (PMC7485978; doi:10.1371/journal.ppat.1008745)
Supplement: S5 Table — (PDF) [file ppat.1008745.s013.pdf]

S5 Table. Strains and plasmids used in this study

Strain or plasmid	Relevant genotype	Source
E. coli strains		
DH5-α λpir	F’ *endA1 hsdR17 supE44 thi-1 recA1 gyrA96 relA1* Δ(*argF-lacZYA*) U169(Φ80*lac*D*M15*) λ pir	BCCM Gene Corner
CC118 λ *pir*	Δ(*ara-leu*) *araD* Δ*lac*X74 *galE galK phoA20 thi-1 rpsE rpoB argE*(*Am*) *recA1* λ *pir*	(Herrero et al., 1990)
S17-1 λ *pir*	Tpr Smr recA, thi, pro, rK- mK+ RP4:2-Tc:MuKm Tn7 *λ pir*	(de Lorenzo and Timmis, 1994)
V. cholerae strains		
FY_Vc_2	Rugose variant, *V. cholerae* O1 El Tor A1552, Rif^r	(Yildiz and Schoolnik, 1999)
FY_Vc_4327	Δ*vps-I*Δ*vps-II*	(Fong et al., 2010)
FY_Vc_3679	Δ*vpsU*	(Fong et al., 2010)
FY_Vc_3384	Δ*vpsO*	(Fong et al., 2010)
FY_Vc_9329	Δ*vpsU*Δ*vpsO*	This study
FY_Vc_11467	FY_Vc3679_Δ*vpsU* Tn7::native promoter-vpsU, Gm^r	This study
FY_Vc_13255	FY_Vc3384_ Δ*vpsO* Tn7::lacI promoter from pMalc-5X-vpsO, Gm^r	This study
FY_Vc_11564	*vpsU-C12S*	This study
FY_Vc_11907	*vpsO-K551A*	This study
FY_Vc_13068	FY_Vc_3679 *vpsO-K551A*	This study
FY_Vc_11629	FY_Vc_11512_RΔ*ctxAB*Δ*vpsO* pBAD B *vpsO-myc/his*, Rif^r, Amp^r	This study
FY_Vc_11619	*vpsO-Y727F*	This study
FY_Vc_12022	*vpsO-Y717F, Y723F*	This study
FY_Vc_13398	*vpsO-Y717F, Y720F, Y721F*	This study
FY_Vc_13369	*vpsO-Y723F, Y726F, Y727F*	This study
FY_Vc_11892	*vpsO-Y720F, Y721F, Y726F, Y727F*	This study
FY_Vc_12031	*vpsO-Y72F*	This study
FY_Vc_12815	*vpsO-Y72F, Y150F, Y285F*	This study
FY_Vc_12230	*vpsO-E148A, R151A, R154A*	This study
FY_Vc_12236	*vpsO-E519A, R522A, R525A*	This study
FY_Vc_11589	*vpsO-Y717F*	This study
FY_Vc_11592	*vpsO-Y720F*	This study
FY_Vc_11610	*vpsO-Y721F*	This study
FY_Vc_11613	*vpsO-Y723F*	This study
FY_Vc_11616	*vpsO-Y726F*	This study
FY_Vc_13366	*vpsO-Y720F, Y721F*	This study
FY_Vc_13395	*vpsO-Y726F, Y727F*	This study

FY_Vc_12034	*vpsO-Y150F*	This study
FY_Vc_12037	*vpsO-Y285F*	This study
FY_Vc_12440	*vpsO-Y150F, Y285F*	This study
FY_Vc_12609	*vpsO-Y72F, Y150F*	This study
FY_Vc_12995	*vpsO-Y72F, Y285F*	This study
FY_Vc_1	*V. cholerae* O1 El Tor A1552, Rif^r	(Yildiz and Schoolnik, 1999)
FY_Vc_15677	Δ*vpsO*	This study
FY_Vc_15692	*vpsO*-K551A	This study
FY_Vc_6271	Δ*vpsU* pBAD B *vpsU-myc/his*	This study
FY_Vc_10767	Δ*vpsA* pBAD B-*vpsA-myc/his*	This study
FY_Vc_10771	Δ*vpsB* pBAD B-*vpsB-myc/his*	This study
FY_Vc_6274	Δ*vpsC* pBAD B-*vpsC-myc/his*	This study
FY_Vc_10777	Δ*vpsD* pBAD B-*vpsD-myc/his*	This study
FY_Vc_10781	Δ*vpsE* pBAD B-*vpsE-myc/his*	This study
FY_Vc_10785	Δ*vpsF* pBAD B-*vpsF-myc/his*	This study
FY_Vc_6282	Δ*vpsG* pBAD B-*vpsG-myc/his*	This study
FY_Vc_10791	Δ*vpsH* pBAD B-*vpsH-myc/his*	This study
FY_Vc_10795	Δ*vpsI* pBAD B-*vpsI-myc/his*	This study
FY_Vc_10799	Δ*vpsJ* pBAD B-*vpsJ-myc/his*	This study
FY_Vc_10803	Δ*vpsK* pBAD B-*vpsK-myc/his*	This study
FY_Vc_10807	Δ*vpsL* pBAD B-*vpsL-myc/his*	This study
FY_Vc_10811	Δ*vpsM* pBAD B *vpsM-myc/his*	This study
FY_Vc_10826	Δ*vpsN* pBAD B-*vpsN-myc/his*	This study
FY_Vc_10830	Δ*vpsO* pBAD B-*vpsO-myc/his*	This study
FY_Vc_10834	Δ*vpsP* pBAD B-*vpsP-myc/his*	This study
FY_Vc_14088	Δ*vpsQ* pBAD B-*vpsQ-myc/his*	This study
FY_Vc_240	FY_Vc_2_ Tn7::gfp	(Beyhan and Yildiz, 2007)
FY_Vc_6230	FY_Vc_3679_Tn7::gfp	This study
KDV1276	Rugose pNUT542	This study
KDV1277	Δ*vpsO* pNUT542	This study
KDV1278	Δ*vpsU* pNUT542	This study
KDV1279	*vpsO-K551A* pNUT542	This study
KDV1280	*vpsU-C12S* pNUT542	This study
KDV1281	*vpsO-Y727F* pNUT542	This study
KDV1282	*vpsO-Y720F, Y721F, Y726F, Y727F* pNUT542	This study
KDV1283	*vpsO-Y717F, Y720F, Y721F* pNUT542	This study
KDV1284	*vpsO-Y723F, Y726F, Y727F* pNUT542	This study
KDV1285	*vpsO-Y72F* pNUT542	This study
KDV1286	*vpsO-Y72F, Y150F, Y285F* pNUT542	This study
KDV1287	*vpsO-E148A, R151A, R154A* pNUT542	This study
KDV1288	*vpsO-E519A, R522A, R525A* pNUT542	This study
FY_8902	FY_Vc_2_pMMB67EH	(Giglio et al., 2013)

FY_15745	FY_Vc_3384_pMMB67EH	This study
FY_15747	FY_Vc_3384_pMMB67EH-*vpsO*	This study
Plasmids		
p2GT	pET vector, IPTG-inducible vector for expression of recombinant proteins with N-terminal His6-GST tags, cleavable by TEV protease, Amp^r	QB3 MacroLab
pFY_4443	p2GT-*vpsU*	This study
pFY_4556	p2GT-*vpsU-C12S*	This study
pFY_5813	p2GT-*vpsO*^{473}	This study
pGEV	pGEX-4T vector with an engineered TEV protease site, IPTG-inducible vector for expression of recombinant proteins with N-terminal GST tag, cleavable by TEV protease, Amp^r	GE Healthcare
pFY_5484	pGEV-*vpsO*^{503}	This study
pFY_5495	pGEV- *vpsO*^{503}*-K551A*	This study
pFY_ 5473	pGEV- *vpsO*^{503}*-Y717A, Y720A, Y721A, Y723A, Y726A, Y727A*	This study
pFY_ 4758	pGEV- *vpsO*^{503}*-Y720A, Y721A, Y726A, Y727A*	This study
pGP704sacB28	pGP704 derivative, mob/oriT sacB, Amp^r	G. Schoolnik
pFY_4773	pGP-*vpsU-C12S*, for chromosomal point mutation	This study
pFY_4505	pGP-*vpsO-K551A*, for chromosomal point mutation	This study
pFY_5469	pGP-*vpsO-Y727F*, for chromosomal point mutation	This study
pFY_4520	pGP-*vpsO-Y717F, Y723F*, for chromosomal point mutation	This study
pFY_5516	pGP-*vpsO-Y717F, Y720F, Y721F*, for chromosomal point mutation	This study
pFY_5509	pGP-*vpsO-Y723F, Y726F, Y727F*, for chromosomal point mutation	This study
pFY_4500	pGP-*vpsO-Y720F, Y721F, Y726F, Y727F*, for chromosomal point mutation	This study
pFY_4516	pGP-*vpsO-Y72F*, for chromosomal point mutation	This study
pFY_5068	pGP-*vpsO-Y72F, Y150F, Y285F*, for chromosomal point mutation	This study
pFY_4595	pGP-*vpsO-E148A, R151A, R154A*, for chromosomal point mutation	This study
pFY_4597	pGP-*vpsO-E519A, R522A, R525A*, for	This study

	chromosomal point mutation	
pFY_1434	pMAL-c5X, IPTG-inducible vector designed to produce maltose-binding protein fusions, Ampr	New England Biolabs
pFY_5472	pGP-*vpsO-Y717F*, for chromosomal point mutation, Ampr	This study
pFY_5467	pGP-*vpsO-Y720F*, for chromosomal point mutation, Ampr	This study
pFY_5468	pGP-*vpsO-Y721F*, for chromosomal point mutation, Ampr	This study
pFY_5471	pGP-*vpsO-Y723F*, for chromosomal point mutation, Ampr	This study
pFY_5470	pGP-*vpsO-Y726F*, for chromosomal point mutation, Ampr	This study
pFY_5508	pGP-*vpsO-Y720F, Y721F* , for chromosomal point mutation, Ampr	This study
pFY_5515	pGP-*vpsO-Y726F, Y727F* , for chromosomal point mutation, Ampr	This study
pFY_4510	pGP-*vpsO-Y150F*, for chromosomal point mutation, Ampr	This study
pFY_4512	pGP-*vpsO-Y285F*, for chromosomal point mutation, Ampr	This study
pFY_4615	pGP-*vpsO-Y150F, Y285F*, for chromosomal point mutation, Ampr	This study
pFY_4613	pGP-*vpsO-Y72F, Y150F* , for chromosomal point mutation, Ampr	This study
pFY_4617	pGP-*vpsO-Y72F, Y285F*, for chromosomal point mutation, Ampr	This study
pNUT542	P_{tac}_*sfgfp* expression plasmid, Gm^r	(Fong et al., 2017)

Berk, V., Fong, J.C.N., Dempsey, G.T., Develioglu, O.N., Zhuang, X., Liphardt, J., Yildiz, F.H., and Chu, S. (2012). Molecular architecture and assembly principles of Vibrio cholerae biofilms. Science *337*, 236–239.

Beyhan, S., and Yildiz, F.H. (2007). Smooth to rugose phase variation in Vibrio cholerae can be mediated by a single nucleotide change that targets c-di-GMP signalling pathway. Mol. Microbiol. *63*, 995–1007.

de Lorenzo, V., and Timmis, K.N. (1994). Analysis and construction of stable phenotypes in gram-negative bacteria with Tn5- and Tn10-derived minitransposons. Meth. Enzymol. *235*, 386–405.

Fong, J.C.N., Syed, K.A., Klose, K.E., and Yildiz, F.H. (2010). Role of Vibrio polysaccharide (vps) genes in VPS production, biofilm formation and Vibrio

cholerae pathogenesis. Microbiology (Reading, Engl.) *156*, 2757–2769.

Fong, J.C., Rogers, A., Michael, A.K., Parsley, N.C., Cornell, W.-C., Lin, Y.-C., Singh, P.K., Hartmann, R., Drescher, K., Vinogradov, E., et al. (2017). Structural dynamics of RbmA governs plasticity of Vibrio cholerae biofilms. Elife *6*, e1002210.

Giglio, K.M., Fong, J.C., Yildiz, F.H., and Sondermann, H. (2013). Structural basis for biofilm formation via the Vibrio cholerae matrix protein RbmA. J. Bacteriol.

Herrero, M., de Lorenzo, V., and Timmis, K.N. (1990). Transposon vectors containing non-antibiotic resistance selection markers for cloning and stable chromosomal insertion of foreign genes in gram-negative bacteria. J. Bacteriol. *172*, 6557–6567.

Yildiz, F.H., and Schoolnik, G.K. (1999). Vibrio cholerae O1 El Tor: identification of a gene cluster required for the rugose colony type, exopolysaccharide production, chlorine resistance, and biofilm formation. Proc. Natl. Acad. Sci. U.S.a. *96*, 4028–4033.
